# Asymmetric connectivity of spawning aggregations of a commercially important marine fish using a multidisciplinary approach

**DOI:** 10.7717/peerj.511

**Published:** 2014-08-07

**Authors:** Adrian Munguia-Vega, Alexis Jackson, Silvio Guido Marinone, Brad Erisman, Marcia Moreno-Baez, Alfredo Girón-Nava, Tad Pfister, Octavio Aburto-Oropeza, Jorge Torre

**Affiliations:** 1 PANGAS Science Coordination, Comunidad y Biodiversidad A.C., Guaymas, Sonora, México; 2 Conservation Genetics Laboratory, School of Natural Resources and the Environment, The University of Arizona, Tucson, AZ, USA; 3 Department of Ecology and Evolutionary Biology, University of California Santa Cruz, Santa Cruz, CA, USA; 4 Departamento de Oceanografía Física, Centro de Investigación Científica y de Educación Superior de Ensenada, Ensenada, Baja California, México; 5 Marine Biology Research Division, Scripps Institution of Oceanography, University of California, San Diego, La Jolla, CA, USA; 6 Universidad Autónoma de Baja California, Ensenada, Baja California, México; 7 School of Natural Resources and the Environment, Center for Latin American Studies, The University of Arizona, Tucson, AZ, USA

**Keywords:** Biophysical models, Population genetics, Oceanography, Gulf of California, Fisheries, No-take zones, Marine reserves, Larval dispersal, Marine connectivity

## Abstract

Understanding patterns of larval dispersal is key in determining whether no-take marine reserves are self-sustaining, what will be protected inside reserves and where the benefits of reserves will be observed. We followed a multidisciplinary approach that merged detailed descriptions of fishing zones and spawning time at 17 sites distributed in the Midriff Island region of the Gulf of California with a biophysical oceanographic model that simulated larval transport at Pelagic Larval Duration (PLD) 14, 21 and 28 days for the most common and targeted predatory reef fish, (leopard grouper *Mycteroperca rosacea*). We tested the hypothesis that source–sink larval metapopulation dynamics describing the direction and frequency of larval dispersal according to an oceanographic model can help to explain empirical genetic data. We described modeled metapopulation dynamics using graph theory and employed empirical sequence data from a subset of 11 sites at two mitochondrial genes to verify the model predictions based on patterns of genetic diversity within sites and genetic structure between sites. We employed a population graph describing a network of genetic relationships among sites and contrasted it against modeled networks. While our results failed to explain genetic diversity within sites, they confirmed that ocean models summarized via graph and adjacency distances over modeled networks can explain seemingly chaotic patterns of genetic structure between sites. Empirical and modeled networks showed significant similarities in the clustering coefficients of each site and adjacency matrices between sites. Most of the connectivity patterns observed towards downstream sites (Sonora coast) were strictly asymmetric, while those between upstream sites (Baja and the Midriffs) were symmetric. The best-supported gene flow model and analyses of modularity of the modeled networks confirmed a pulse of larvae from the Baja Peninsula, across the Midriff Island region and towards the Sonoran coastline that acts like a larval sink, in agreement with the cyclonic gyre (anti-clockwise) present at the peak of spawning (May–June). Our approach provided a mechanistic explanation of the location of fishing zones: most of the largest areas where fishing takes place seem to be sustained simultaneously by high levels of local retention, contribution of larvae from upstream sites and oceanographic patterns that concentrate larval density from all over the region. The general asymmetry in marine connectivity observed highlights that benefits from reserves are biased towards particular directions, that no-take areas need to be located upstream of targeted fishing zones, and that some fishing localities might not directly benefit from avoiding fishing within reserves located adjacent to their communities. We discuss the implications of marine connectivity for the current network of marine protected areas and no-take zones, and identify ways of improving it.

## Introduction

Knowledge of patterns of larval dispersal is essential to implement fully-protected marine reserves (no-take zones), a tool frequently used to enhance the conservation of biodiversity and the recovery of fisheries (Gaines et al., 2010). Reserves must either be large enough to be self-sufficient via local retention (larvae retained or returning to the reserve where they were produced) (Toonen et al., 2013), or need to be linked by a network of reserves for persistence (larval supply from reserves to other reserves/fished sites) (Hastings & Botsford, 2006; White et al., 2010a). However, the efficacy of networks of reserves has been hindered by a lack of knowledge regarding complex patterns of marine connectivity (Sale et al., 2005; Burgess et al., 2014). A multidisciplinary approach could best address the intricacy of connectivity by merging biophysical models of ocean currents that generate connectivity hypotheses using detailed biological information on the spatial and temporal distribution of propagules (larvae), followed by validation with empirical population genetics data (Gilg & Hilbish, 2003; Baums, Paris & Cherubin, 2006; White et al., 2010b; Alberto et al., 2011; Crandall, Treml & Barber, 2012; Foster et al., 2012; Soria et al., 2012; Feutry et al., 2013). Use of multiple methodologies could also help advance an increasing interest in incorporating genetic information into marine spatial planning, for example, by identifying sites with high genetic diversity that hold evolutionary potential under future environmental change (Beger et al., 2014).

Marine connectivity within a single species is influenced by multiple biological and physical factors including: spawning time and location, pelagic larval duration (PLD), their interaction with ocean current speed and direction, as well as the distribution of suitable habitat for settlement (reviewed by Cowen & Sponaugle, 2009). Additionally, many commercially exploited species of invertebrates and fishes display metapopulations that are connected via larval dispersal (Cowen et al., 2000). Few attempts have been done to establish multidisciplinary approaches to understand marine connectivity (e.g., Soria et al., 2012; Soria et al., 2014). A challenge is to find a key species that can be a relevant case study, for which this information can be gathered relatively easily, and can be used as an umbrella species to design marine reserves, although this approach might not be always effective (Bird et al., 2007; Toonen et al., 2011).

The Midriff Islands region includes 45 islands and islets located on the division between the northern and central Gulf of California (GC). The bathymetry of this region constitutes one of the most notable features of the GC, which is formed by a series of deep basins (e.g., Dolphin, Salsipuedes, San Pedro Martir) and sills, and which restrict the circulation between the northern and central GC (Hernandez-Ayon et al., 2013). The combination of this restricted circulation, with the weakening of stratification by the turbulent kinetic energy released during the passage of the semidiurnal tidal wave through the constrictions of this archipelago (Argote et al., 1995) results in an oceanographically complex body of water (Lavin & Marinone, 2003) and the presence of a surface thermal front (Paden & Abbott, 1991). These features have been documented to restrict larval distributions, generating differences between northern and central fish larval assemblages (Danell-Jiménez et al., 2009). This study was centered in the Northern GC, which is characterized by a large-scale seasonally reversing gyre that has been documented by different approaches, including satellite tracked drifters (Lavin et al., 1997), geostrophic calculations (Carrillo & Palacios-Hernández, 2002), current meters (Palacios-Hernandez et al., 2002) and numerical models (Marinone, 2003; Marinone, 2012). The basin-wide gyre is cyclonic (anti-clockwise) from May to September (Lavin et al., 1997), and reverses to an anti-cyclonic (clockwise) gyre from October to March. The forcing systems have been related to the annual-period monsoonal winds, the Pacific Ocean and the superficial heat flux (Beier & Ripa, 1999).

**Figure 1:**
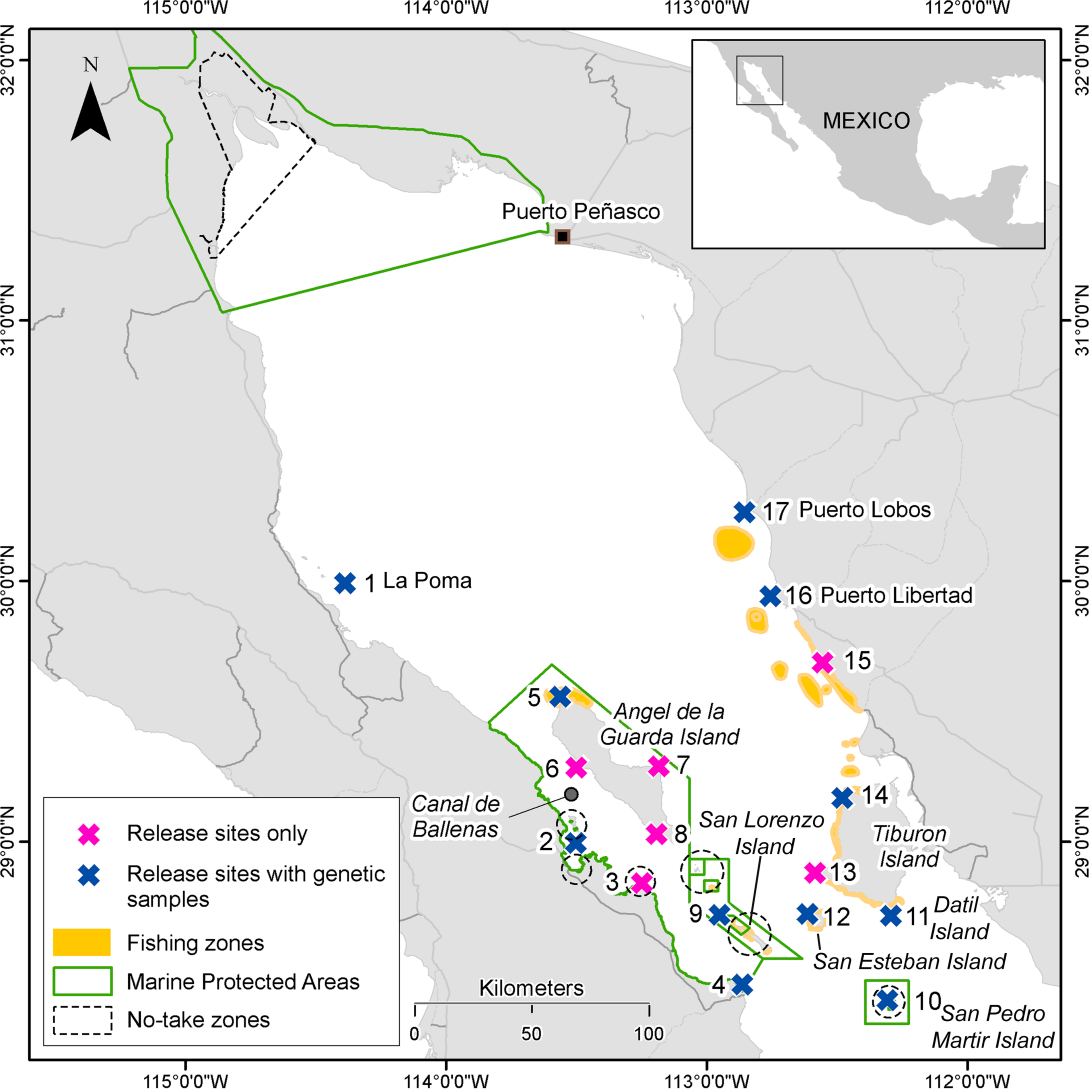
Area of study around the Midriff Islands in the Gulf of California. Study sites, in which modeled and genetic connectivity was measured (release sites with genetic samples) and those only for modeled connectivity (release sites only), including marine protected areas, no-take zones and fishing zones identified by interviews with fishers (Moreno-Baez et al., 2010; Moreno-Báez et al., 2012). Note that no-take zones are small and dotted circles denote approximate location but not size.

Small fisheries worldwide comprise most of the global catch, yet most lack formal assessments and are continuing to decline (Costello et al., 2012). The leopard grouper (*Mycteroperca rosacea*, Streets 1877) is the most common and heavily targeted grouper by small-scale and recreational fisheries in the GC (Sala et al., 2003; Craig, Sadovy de Mitcheson & Heemstra, 2011). Due to increased fishing pressure and observed declines in fisheries landings, sizes of harvested fish, and population abundances in some areas of the GC over the past few decades (Sala et al., 2004), the International Union for the Conservation of Nature (IUCN) currently lists *M. rosacea* as ‘Vulnerable’ (Craig & Sadovy, 2008). While commercial fishers are required to hold a finfish permit and record their landings of leopard grouper to their local fisheries offices, no specific regulations related to catch, size, or gear restrictions exist for this species. Currently, all *M. rosacea* catches are aggregated in a large group, registered as “finfish” with other 270 fish species according to the National Fisheries Chart (Anonymous, 2012). Marine Protected Areas (MPAs) represent the primary, current conservation and management strategy that has been implemented for this or any other reef fish in the GC (Cudney-Bueno et al., 2009; Aburto-Oropeza et al., 2011), and are mainly concentrated in the western coast of the GC and contain a few small no-take zones (Fig. 1).

Our main hypothesis is that source–sink larval metapopulation dynamics in the Midriff Island region, including the direction and frequency of larval dispersal described according to a numerical oceanographic model can help to significantly explain empirical genetic patterns and inform sustainable fisheries and the design of a network of marine reserves. We searched for agreement between models and empirical data at two levels: node-based analyses including genetic diversity within sites and matrix-based analyses including genetic structure between sites. To achieve this, we first determined the distinct spawning season for leopard grouper and identified the spatial distribution of spawning aggregation sites and fishing zones across the entire region. We modeled connectivity with a biophysical model and used graph theory to describe metapopulation dynamics. We contrasted distinct measures describing larval dispersal derived from graph theory against empirical estimates of genetic diversity and differentiation to corroborate model expectations. Our approach also aimed to identify sites that are likely self-sustaining and important sources and sinks for leopard grouper larvae, including locations that may lie inside or outside the borders of existing MPAs. Results of this study provide insights on validating biophysical models with empirical genetic data, and also on the benefits and limitations of the current network of MPAs to fishing communities in the Midriffs region that harvest *M. rosacea*. This work will help identify areas that may serve as ideal locations for refuges of spawning adults or juveniles of this economically important yet vulnerable species.

## Methods

### Spawning sites, season and period

*Mycteroperca rosacea* is a large predatory reef fish (Teleostei: Epinephelidae) endemic to the GC bioregion. It ranges from Bahía Magdalena in the Pacific coast of the Baja California Peninsula south to Bahía Banderas in Nayarit, Mexico, including all rocky-reefs within the interior of GC (Thomson, Findley & Kerstitch, 2000; Robertson & Cramer, 2009; Hastings, Findley & Van der heiden, 2010). Ecologically, it represents the most common and numerically abundant fish top predator on reefs in the entire GC. Individuals can reach 1 m in length and at least 22 years of age (Diaz-Uribe, Elorduy-Garay & Gonzalez-Valdovinos, 2001). Spawning occurs in the evening within groups of 6–40 individuals and is not correlated with the lunar cycle (Erisman, Buckhorn & Hastings, 2007). Histological and population data indicate gonochorism, with no evidence of post-maturational sex change found in adults caught in the wild (Erisman, Rosales-Casián & Hastings, 2007).

We used information from underwater and fisheries surveys conducted at locations throughout the Midriffs Islands region in the GC, Mexico (Fig. 1). This information helped to identify the sites to simulate the dispersal of leopard grouper eggs and larvae from spawning aggregation sites. Underwater surveys were performed at 33 sites throughout the Midriffs during the spawning season of *M. rosacea* (April–June) in 2008, 2009, and 2010. Evidence of the formation of spawning aggregations were based on standard protocols (Colin, Sadovy & Domeier, 2003) and those adapted for leopard grouper (Erisman, Rosales-Casián & Hastings, 2007). Direct evidence of spawning aggregations included observations of courtship or spawning behavior or the collection of females with hydrated or ovulated oocytes. Indirect evidence involved observations of putative females with enlarged abdomens indicative of imminent spawning, color patterns associated with courtship, the collection of males with ripe testes, and abundances and densities of fish that were markedly higher (e.g., 3-fold increases or greater) than observed during non-spawning months. Additional indirect evidence of spawning aggregations was acquired through interviews with commercial fishers at five fishing communities (Bahía de los Ángeles, Bahía de Kino, Desemboque Seri, Puerto Libertad and Punta Chueca) during 2005 and 2006 (Moreno-Baez et al., 2010; Moreno-Báez et al., 2012).

While the general spawning season for *M. rosacea* in the GC occurs from late April to June (Erisman, Buckhorn & Hastings, 2007), it was necessary to collect empirical data to narrow the specific spawning season from the Midriff Islands region. We acquired gonad samples of adult female leopard groupers (i.e., >30 cm TL; Erisman, Buckhorn & Hastings, 2007) from commercial fishers on a monthly basis from December 2008 to June 2010. Fish were captured by gill nets or handlines at various sites at or near San Pedro Martir and Tiburon islands, and Bahia Kino (Fig. 1). We processed tissue taken from the central portion from one gonad lobe for each sample using standard histological techniques (Humason, 1972) in order to determine sex and developmental stage. Classes of ovarian and testicular development were adapted from previous studies (Erisman, Rosales-Casián & Hastings, 2007), and stages of gametogenesis followed previously established definitions (Wallace & Selman, 1981).

We determined the duration of the spawning season using a combination of two methods. First, we examined the histological preparations of all gonad samples to identify the percentage of females capable of spawning or actively spawning during each month of sampling. Females categorized as ‘spawning capable’ included those with ovaries dominated by oocytes in advanced stages of vitellogenesis (e.g., primary to tertiary yolk stage), whereas those categorized as ‘actively spawning’ contained ovaries with oocytes in the migratory nucleus, hydrated, or ovulated stage or post-ovulatory follicles were present. Data were pooled by month to estimate the monthly proportion of spawning females over a calendar year. Collection dates of females classified as actively spawning based on histology were used as indicators to determine exact dates of spawning. A second estimate of the spawning season was obtained by calculating the mean monthly gonadosomatic index (GSI = 100 ∗ gonad weight/total body weight) of female *M. rosacea* over the study period. Changes in monthly GSI were used to assess reproductive activity, associating elevated levels with gonadal development and spawning. This information was used in the release dates for larvae in the oceanographic model (see below).

### Supporting fishing knowledge

The central component to documenting the fishing grounds for the *M. rosacea* was captured through a series of interviews implemented across 17 fishing communities in the northern GC (Moreno-Baez et al., 2010; Moreno-Báez et al., 2012). The methodology entailed aggregating local knowledge of a representative set of individual fishers (captains) through semi-structured interviews conducted between December 2005 and July 2006 regarding what, where, when and how they fish. The interview included questions regarding the spatial and temporal distribution of fishing activities but also, their knowledge about locations of spawning aggregations and juvenile habitat. The maps were digitized, georeferenced, and integrated into a geographic information systems (GIS) using ArcGIS 9.2 (ESRI, 1999–2008).

These interviews indicated that fishing activity frequently overlapped spatially with spawning aggregation sites in three main regions: (1) the north end of Angel de la Guarda Island; (2) on the south, western and northern edge of Tiburon Island, and on sites in mainland Sonora north of Tiburon, around Las Cuevitas, Puerto Libertad and Puerto Lobos (yellow areas Fig. 1). Other important fishing zones were identified around Puerto Peñasco in northern Sonora. According to the interviews, the principal fishing season starts in November and ends in June.

### Oceanographic model

Spatial units (Fig. S1) were established to evaluate spatial connectivity by combining physical and political boundaries, as well as local knowledge from fishers (Moreno-Baez et al., 2010; Moreno-Báez et al., 2012). We incorporated coastline and bathymetry developed by the National Geophysical Data Center (http://www.ngdc.noaa.gov/mgg/shorelines/shorelines.html) and the marine protected areas in Mexico (www.conanp.gob.mx), respectively. We used the spatial union function to integrate the different boundaries and define the spatial units, under ArcGIS 10.1 (ESRI) with the Spatial Analyst Extension and Model Builder tools. The size of the spatial units varied from 13 to 812 km^2^ (Fig. S1).

In a computer simulation exercise, four thousand particles were released at a depth of 5 m at each of 17 spawning aggregation sites (Fig. 1). Particles were tracked for 28 days, which is close to the maximum PLD for *M. rosacea* (Aburto-Oropeza et al., 2007). We released particles during eight dates representing the four neap and four spring tides during May and June (Fig. S2), which based on our observations covers the peak spawning period around the Midriff Islands region (see Results). Since seasonal oceanographic regimes are consistent across years in the GC (Marinone, 2003; Soria et al., 2014), the simulation year (2007) was chosen arbitrarily, and the phase of the spring-neap cycle is simply shifted from year to year.

We used the velocity field from the GC implementation of the three-dimensional baroclinic Hamburg Shelf Ocean Model (HAMSOM) (Backhaus, 1985) to calculate the particle trajectories. The model has been described in detail for the GC (Marinone, 2003; Marinone, 2008). Its domain has a mesh size of 2.5′/3 × 2.5′/3 (∼1.31 × 1.54 km) in the horizontal and 12 layers in the vertical with nominal lower levels at 10, 20, 30, 60, 100, 150, 200, 250, 350, 600, 1,000 and 4,000 m. The model equations are solved semi-implicitly with fully prognostic temperature and salinity fields, thus allowing time-dependent baroclinic motions. The model is started from rest with a 300 s time step and becomes periodically stable after three years. Results for this study were obtained from the fourth year of the model when it adequately reflects the main seasonal signals of surface temperature, heat balance, tidal elevation and tidal currents and surface circulation in the NGC (Lavin et al., 1997; Marinone, 2003). The forcing includes at the open boundary model tidal components (M2, S2, N2, K2, K1, O1, P1, Ssa, and the Sa), climatological hydrography historical data and at the sea surface climatological heat and fresh water fluxes. We used the seasonal climatology constructed from QUICKSCAT data as forcing for wind. The Lagrangian trajectories are due to the Eulerian velocity field plus a random-walk contribution related to turbulent eddy diffusion processes (Visser, 1997; Proehl et al., 2005). We obtained values of the diffusivities from the numerical model. A pseudo-advective term was introduced, since the vertical diffusivity is not constant, to prevent particles from walking away from areas of high to low diffusivities. The velocity at each particle position and the vertical eddy coefficients are calculated by bilinear interpolation of the instantaneous Eulerian velocity fields and the eddy coefficient from the numerical model, which were saved every hour. The horizontal diffusivity is taken as a constant (100 m^2^/s). We assumed that larvae are advected as passive particles and do not migrate vertically downward to deep depths, as in other studies (e.g., Watson et al., 2010), given that leopard grouper recruit to shallow *Sargassum spp.* beds of <5 m deep (Aburto-Oropeza et al., 2007).

### Modeled connectivity

Hourly latitude and longitude data for each modeled particle were imported into MatLab (MATHWORKS). We estimated connectivity at different time intervals: 336 h (14 days), 504 h (21 days) and 672 h (28 days) respectively after the release dates. These PLDs were selected based on the average time of flexion in groupers that corresponds to the onset of larval behavior (14 days) (Cowen, 2002; Gracia-Lopez et al., 2005) and the maximum PLD (28 days) (Aburto-Oropeza et al., 2007). To identify the intersection between particles and the recruitment areas (spatial units), we used a selection by location function (in-polygon). We then generated connectivity matrices using the proportion of larvae that settled at each location relative to the total number of larvae released at each site. We constructed matrices averaging for the eight spawning dates within each PLD (i.e., days 14, 21, 28). The probability of local retention (i.e., diagonal in the connectivity matrix) was calculated as the proportion of particles produced locally that remained within the spatial unit at the end of the PLD (Burgess et al., 2014). The probabilities within each site were summarized with two statistics aimed at describing source–sink dynamics. Export probability was defined as the proportion of larvae produced within a site that successfully settled within any of the other 16 coastal areas at the end of the PLD. Import probability was defined as the proportion of all larvae produced among the 17 sites that settled within each site. This later metric is identical to self-recruitment as defined by Burgess et al. (2014).

Marine connectivity patterns were displayed using graph theory and a spatial network approach (Treml et al., 2008; Treml et al., 2012) with the software NODEXL (Smith et al., 2010). We calculated five statistics that describe the relationships among elements (i.e., sites or nodes) in complex networks (Newman, 2003), including: (1) graph size (the total number of directed links within a graph); (2) in-degree (number of links that enter a node); (3) out-degree (number of links that leave a node); (4) betweenness centrality, or the proportion of shortest paths between all node pairs that pass through a particular node; and (5) clustering coefficient, a measure of how close the neighbors of a node are to being a clique (complete graph). Betweenness centrality can be viewed as a measure of resilience by measuring how many paths will get longer when a node is removed (Newman, 2003). This measure highlights ‘most used’ dispersal pathways or stepping stones that act like gateways through which genes or individuals have to pass to spread to other nodes, emphasizing key sites for multigenerational connectivity (Andrello et al., 2013). The clustering coefficient varies from 1 (when every neighbor connected to the node is also connected to all the other nodes) to zero (if zero neighbors connected to the node are connected to other nodes connected to node in question).

We used the software GENETICSTUDIO (Dyer, 2009) to calculate two distinct types of symmetric matrices describing the distance separating pairs of nodes (sites) according to the topology of the network: (1) adjacency matrix, containing the actual edge weight (probability of larval dispersal, regardless of the direction) in the off-diagonal elements that attach the indexed nodes, while nodes that are not connected are denoted as zero; and (2) graph distance, or the length of the minimum topological distance (i.e., shortest geodesic path) between two nodes, calculated conditional to the entire data set of nodes and edges, and which has statistical properties including homoscedasticity and stability (Dyer, Nason & Garrick, 2010).

### Genetic connectivity

We collected tissue samples from the pectoral fins of *M. rosacea* from 11 sites included in our modeling exercise around the Midriff Islands region (Fig. 1). Samples were acquired in fish markets or directly from fishermen at harbors between 2009 and 2012 under IACUC protocol Berng1101. We interviewed both fish vendors and fishermen to determine the approximate localities where fish were collected. Immediately after collection, samples were stored in 95% ethanol and kept at −20 ° C in the laboratory. Genomic DNA was extracted using standard chloroform extraction protocols (Sambrook, Fritsch & Maniatis, 1989). We amplified a 787 bp fragment of mitochondrial marker cytochrome *b* using primers Gludgl 5′-TGAYTTGAARAACCAYCGTTG-3′ and CB3H 5′-GGCAAATAGGAARTATCATTC-3′ (Palumbi et al., 1991). Thermocycler parameters were as follows: initial hold at 94 °C/5 min, 35 cycles of 94 °C/45 s, 45 °C/45 s, 72 °C/45 s, with a final extension of 72 °C/7 min. We developed species-specific primers for *M. rosacea* (MYCROS Forward: 5′-TTCTCCCACTACCCTGATTC-3′ and MYCROS Reverse: 5′-TACGTAGGCTTGGATCATTG-3′) to amplify a 726 bp fragment of mitochondrial marker ATPase. Thermocycler parameters were as follows: initial hold at 94 °C/5 min, 35 cycles of 94 °C/30 s, 54 °C/30 s, 72 °C/30 s, with a final extension of 72 °C/7 min. After purification of PCR products following ABI manufacturer’s protocols (ABI, Perkin-Elmer), we sequenced clean PCR products on an ABI 3730xl automated sequencer (Applied Biosystems, Foster City, CA).

We calculated molecular diversity indices including nucleotide diversity (*π*) and haplotype diversity (*h*). We corrected haplotype diversity using CONTRIB (Petit, El Mousadik & Pons, 1998) to account for differences in sample size between sites based on rarefaction to a minimum sample size of *n* = 4.

Phylogenetic relationships among sequences were inferred from a haplotype network based on pairwise differences between haplotypes generated using Arlequin (Excoffier, Laval & Schneider, 2005) and R software (R Core Team, 2013). To test for hierarchical population structure we performed an Analysis of Molecular Variance (AMOVA) in Arlequin. AMOVA significance was estimated using a permutation test of 10,000 replicates. The 11 sites were clustered into three regions: Baja Peninsula, the Midriff Islands and the Sonoran coast (Table 2). Chi-squared analyses were concurrently performed using DnaSP version 5.10 (Librado & Rozas, 2009) to test for patterns of regional subdivision. Pairwise *F_ST_* and *ϕ_ST_* values were calculated between each location to assess how close populations are to alternate fixation. While *F_ST_* considers all haplotypes identical and its maximum value decreases as the internal genetic diversity of population samples increases, *ϕ_ST_* effectively groups haplotypes considering their phylogenetic distance and is robust to increased within-populations genetic diversity (reviewed by Bird et al., 2011). When mutation rate is greater than migration rate and there is some restriction to gene flow (i.e., *F_ST_*/*ϕ_ST_* < 1) the evolutionary relationship among haplotypes can provide additional resolution to explain where an haplotype is found among distinct populations (reviewed by Bird et al., 2011). However, *ϕ_ST_* has the potential to introduce noise in cases where migration rate is much greater than mutation rate and the evolutionary identity of individuals haplotypes is unrelated to the geographic location in which they are located (i.e., *F_ST_*/*ϕ_ST_* > 1). We also calculated the standardized index ${F}_{S T}^{{\prime}}$ (Meirmans & Hedrick, 2011) by dividing the original *F_ST_* by the maximum value *F_ST_* can achieve while maintaining the within-population diversity. Maximum values were calculated by recoding the alleles in such a way that every population only contained alleles unique to that population, following Meirmans & Hedrick (2011). In contrast to *F_ST_* and *ϕ_ST_* which are fixation indexes, ${F}_{S T}^{{\prime}}$ measures genetic differentiation and reaches its maximum when no alleles are shared between populations (Bird et al., 2011).

We constructed a population graph from the distribution of haplotype frequencies with the software GENETICSTUDIO. A population graph is an analysis that allows genetic structure to define a graph-theoretic topology by capturing the high dimensional genetic covariance among all nodes considered simultaneously. An edge is placed between nodes that are conditionally dependent of each other, based on the remaining data in the model. With the empirical network, we calculated the same five node-based network metrics in NODEXL, and the adjacency and graph distance matrices (also known as conditional genetic distance, Dyer, Nason & Garrick, 2010) in GENETICSTUDIO as described above for the modeled networks.

To test if the ocean model could help to explain the empirical genetic data, we performed node-based and matrix-based analyses. Node base analyses were done fitting a linear model in R between empirical corrected haplotype diversity and nucleotide diversity within each site using in-degree, out-degree and betweenness centrality estimates from networks at each PLD as explanatory variables. Theory predicts that genetic diversity levels observed within sites is highly dependent upon the amount of migration from source populations (Gaggiotti, 1996), and that genetic diversity increases in sink sites that accumulate larvae and genetic variants from multiple sources (Kool et al., 2011). We also tested for a correlation between modeled and empirical estimates of betweenness centrality and clustering coefficient for each node with a linear regression. We predicted that if model estimates of node centrality depicted an accurate description of the actual connectivity among sites, modeled and empirical values should be significantly correlated.

Matrix-based analyses were done with Mantel tests implemented in the software IBDWS employing 10,000 randomizations (Jensen, Bohonak & Kelley, 2005). First, we tested whether larval dispersal fit a stepping-stone model where gene flow is limited by geographic distance by comparing the pairwise matrices of *ϕ_ST_*, *F_ST_* and ${F}_{S T}^{{\prime}}$ against geographic distances. Second, we predicted that the presence of a larval dispersal link between two nodes and its intensity (probability), as estimated from an adjacency matrix from the modeled networks, would reduce the level of genetic differentiation observed. We conducted a partial Mantel test between the modeled adjacency matrix and the empirical (log) *ϕ_ST_*, (log) *F_ST_* and (log) ${F}_{S T}^{{\prime}}$ matrices, respectively, while controlling for (log) geographic distance. Third, we predicted that, if oceanographic models represent an accurate description of connectivity patterns among sites, then sites connected by larger graph distances between two sites, considering the topology of the modeled network, would have larger levels of genetic structure. We used a partial Mantel test between modeled graph distances and the empirical (log) *ϕ_ST_*, (log) *F_ST_* and (log) ${F}_{S T}^{{\prime}}$ matrices, respectively, while controlling for (log) geographic distances. Fourth, under the same previous hypothesis, we performed a Mantel test comparing the empirical *ϕ_ST_*, *F_ST_* and ${F}_{S T}^{{\prime}}$ matrices, respectively, against the (log) graph distance from the modeled networks. When log-transforming genetic distances, negative values were excluded from the analyses. We predicted that if the modeled matrices were close to reality, then modeled and empirical matrices should be significantly correlated. We tested for a correlation between the modeled and empirical adjacency and graph distance matrices with linear regression analyses. Given the large number of tests performed in the node-base and matrix-based analyses, we corrected the critical *P* value 0.05 with the graphically sharpened method (Benjamini & Hochberg, 2000) to account for a false discovery rate in multiple tests within each analysis before assuming statistical significance.

We evaluated three different larval migration models based on the genetic data using Migrate-n 3.2.16 (Beerli & Palczewski, 2010). First, we tested an unrestricted full migration model between all sampling localities. Next, we considered two models with three population sizes comprised of a subsampling (*n* = 30) from sampling localities in the Baja Peninsula, the Midriff Islands, and the Sonoran coast. One model assessed the hypothesis of unidirectional gene flow from the Baja Peninsula across the Midriff Islands and towards the Sonoran coast, while the other model tested gene flow in the reverse direction. The latter two models reflect seasonal differences in directionality of a cyclonic and anticlyclonic gyres in the northern GC. Using a Bezier approximation, we chose the most appropriate model for our dataset by taking the natural log of the ratio of the marginal likelihoods (Bayes factors) for each model (Beerli & Palczewski, 2010). Running conditions for Migrate-n were as follows: 5,000,000 recorded steps, a burn-in of 2,500,000 steps, a static heating scheme using 20 temperatures, a tree swapping interval of 1, and an upper prior boundary for migration set to 7,500.

To further cross-validate the genetic and modeled connectivity data, we searched for statistical evidence of the presence of modularity or graph structure in the modeled and empirical networks based on the three geographic groups (Baja, Midriffs and Sonora). Analyses were done with GENETICSTUDIO, where we considered each group in a sub-graph one at a time, separated from the other two.

## Results

### Spawning sites, season and period

We identified 17 sites associated with 17 distinct spatial units for the release of virtual larvae in the simulation model based on direct and indirect evidence of the presence of spawning aggregations for leopard grouper (Table 1). Although some spatial units had evidence of multiple spawning aggregations, we assumed their close proximity along with the spatial resolution of the oceanographic model meant multiple aggregations within the same unit would disperse larvae in similar directions. Individual sites were distributed throughout the region and fulfilled a range of 3–7 criteria used to define a spawning aggregation site (Table 1), with an average of 4.76 criteria ± 1.48 (SD). A marked increase in the abundance of adult groupers during the spawning season relative to the non-spawning season was the most common evidence (recorded at all 17 sites). Other types of indirect evidence such as the observation of gravid females with swollen abdomens, observations of fish exhibiting courtship coloration, the collection of running-ripe males, or elevated catch rates by fishers during the spawning season were also recorded for the majority of sites. Direct evidence of spawning via the collection of hydrated females was recorded for 65% of the sites, whereas spawning was observed at only 35% of the sites.

**Table 1:** Study sites in the Gulf of California and selection criteria. Each site was selected based on seven criteria to define where spawning aggregations might act like source of larvae. The order of sites follows the predominant cyclonic (anti-clockwise) circulation in the Midriff Island region during spawning of *M. rosacea*.

Site #	Site name	High abundance of fish	Elevated catch rates	Hydrated females collected	Running-ripe males collected	Courtship or spawning observed	Gravid females observed	Courtship coloration observed	Criteria observed
1	La Poma	✓	✓		✓				3
2	La Ventana	✓	✓	✓	✓	✓	✓	✓	7
3	Chorros	✓		✓	✓		✓	✓	5
4	San Francisquito	✓	✓				✓	✓	4
5	Punta Refugio	✓	✓	✓	✓	✓	✓	✓	7
6	Los Machos	✓		✓	✓	✓	✓	✓	6
7	Punta Diablo	✓				✓	✓	✓	4
8	Punta Roja	✓		✓	✓	✓	✓	✓	6
9	San Lorenzo Island	✓	✓				✓	✓	4
10	San Pedro Martir Island	✓	✓	✓	✓		✓	✓	6
11	Datil Island	✓	✓	✓	✓	✓	✓	✓	7
12	San Esteban Island	✓	✓				✓		3
13	La Tordilla	✓	✓	✓	✓				4
14	El Tecomate	✓	✓	✓	✓				4
15	Las Cuevitas	✓	✓	✓	✓				4
16	Puerto Libertad	✓	✓				✓	✓	4
17	Puerto Lobos	✓	✓	✓	✓				4
	# Sites	17	13	11	12	6	12	11	

**Table 2:** Molecular diversity for each site with genetic samples. For each site, we show their membership to one of three geographic groups, number of samples (*n*), number of haplotypes (*n*H), haplotype diversity (*h*), corrected haplotype diversity (*h*_†_) and nucleotide diversity (*π*).

Location	Group	*n*	*n*H	*h*	*h*_†_	*π*
1. La Poma	Baja	16	12	0.950 ± 0.040	0.920	0.0016 ± 0.0010
2. La Ventana	Baja	52	23	0.870 ± 0.035	0.885	0.0016 ± 0.0010
4. San Francisquito	Baja	23	17	0.949 ± 0.034	0.920	0.0019 ± 0.0012
5. Punta Refugio	Midriffs	25	9	0.840 ± 0.030?	0.872	0.0015 ± 0.0010
9. San Lorenzo Island	Midriffs	11	4	0.673 ± 0.123	0.844	0.0011 ± 0.0008
10. San Pedro Martir Island	Midriffs	17	9	0.860 ± 0.068	0.881	0.0014 ± 0.0010
11. Datil Island	Midriffs	20	13	0.947 ± 0.032	0.919	0.0020 ± 0.0010
12. San Esteban Island	Midriffs	4	4	1.000 ± 0.177	0.943	0.0013 ± 0.0011
14. El Tecomate	Sonora	26	16	0.935 ± 0.034	0.914	0.0019 ± 0.0010
16. Puerto Libertad	Sonora	55	21	0.874 ± 0.033	0.887	0.0016 ± 0.0010
17. Puerto Lobos	Sonora	11	7	0.909 ± 0.066	0.902	0.0018 ± 0.0012

**Figure 2:**
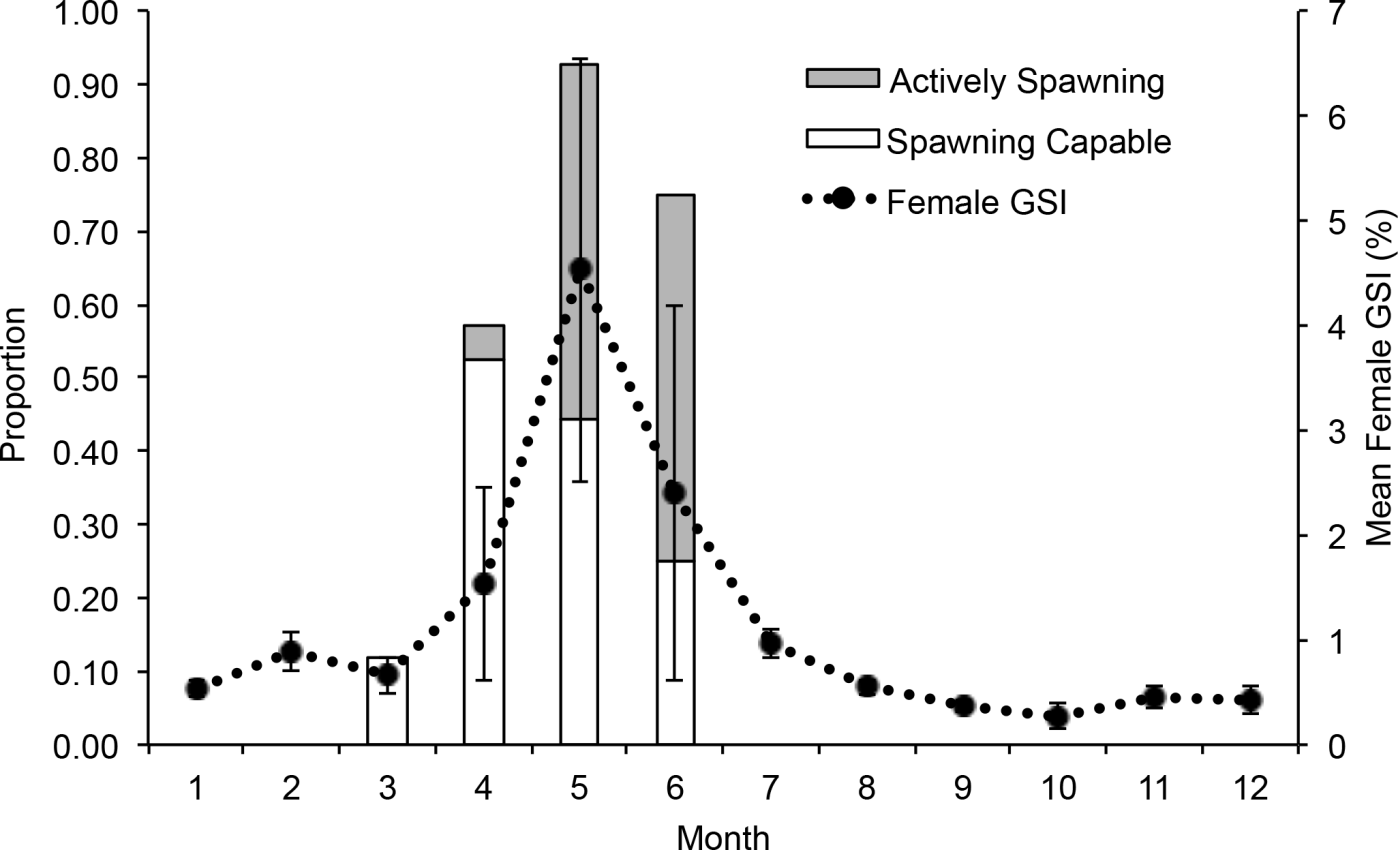
Spawning season and period. Monthly proportion of actively spawning and spawning capable females (left *y* axis) and female gonadosomatic index (GSI, right *y* axis) for the Midriff Islands collected in 2009.

A total of 162 samples of female *M. rosacea* were collected from commercial fishers, with an average of 14 samples collected each month (range = 8–27). Based on microscopic examinations of gonadal tissue samples, females in the spawning capable phase were collected from March through June, and actively spawning females were collected mainly during May and June. The GSI of adult females showed elevated levels from April to June, with a peak during May (Fig. 2). When the results of the gonadal phases and GSI were combined, they indicate that *M. rosacea* spawn from April to June in the Midriffs region, with peak spawning activity occurring in May and June. Actively spawning females were collected on three days in 2009 (14 May, 31 May, 25 June) and two days in 2010 (25 April, 7 May).

### Modeled connectivity

**Figure 3:**
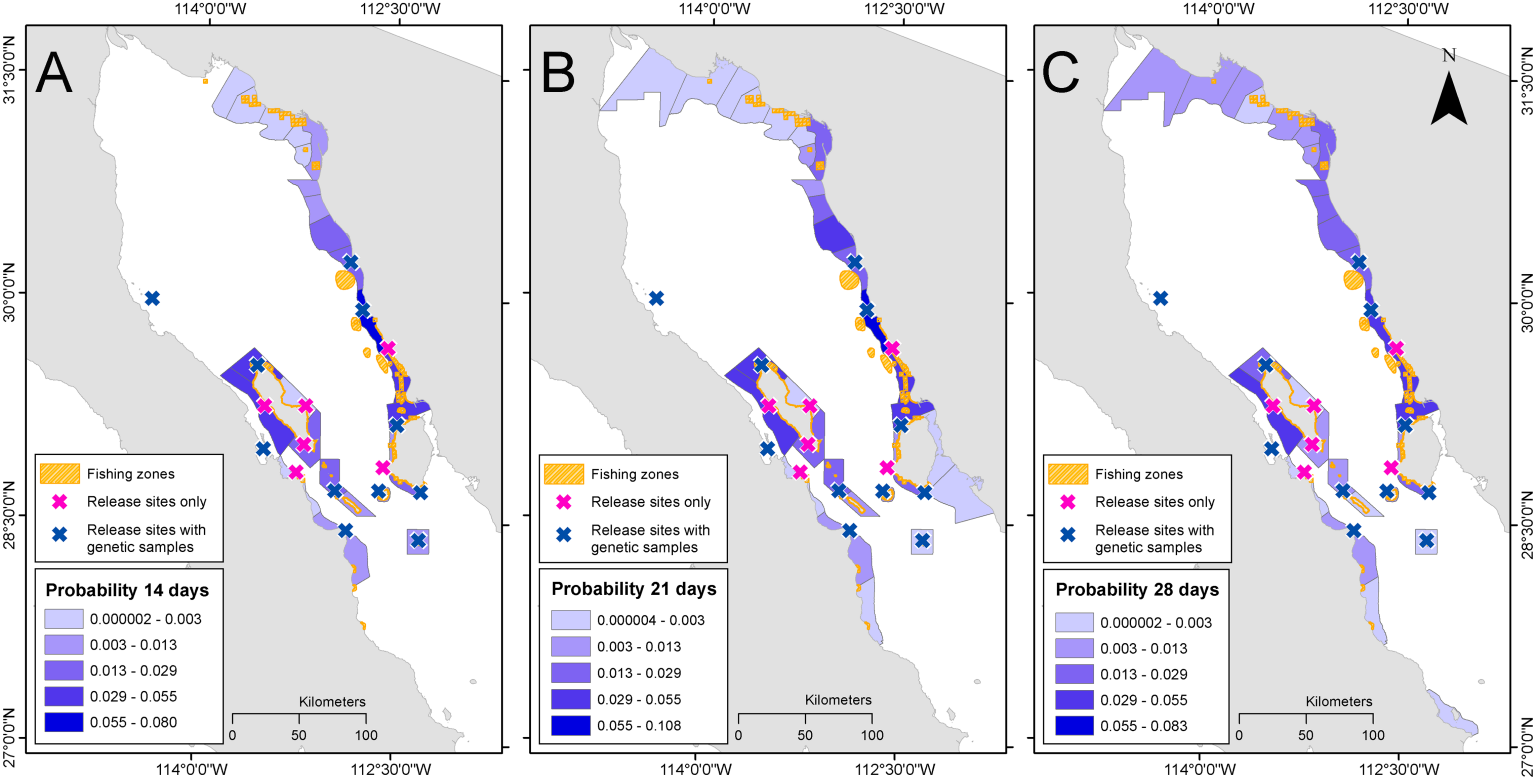
Probability of larval density. Density of all virtual larvae released in coastal areas after PLD 14 days (A), 21 days (B) and 28 days (C) in each coastal spatial unit of analysis.

From all simulated particles released (4,000 particles × 17 sites × eight release dates = 544,000), coastal areas that are suitable for larval recruitment captured 55.13% (PLD 14 days), 48.07% (PLD 21 days) and 42.03% (PLD 28 days). Remaining particles did not reach any coastal habitat by the end of the PLD. Simulations of ocean currents produced the highest concentrations of larvae in: (1) Canal de Ballenas, (2) north end of Angel de la Guarda Island, (3) north end of Tiburon Island, and (4) in coastal areas located to towards the north after PLD 28 days (around Las Cuevitas and Puerto Libertad Fig. 3). Other important areas were located at the south end of Tiburon Island and Puerto Lobos. Simulations at PLD 14 and 21 days indicated similar trends (Fig. 3).

**Figure 4:**
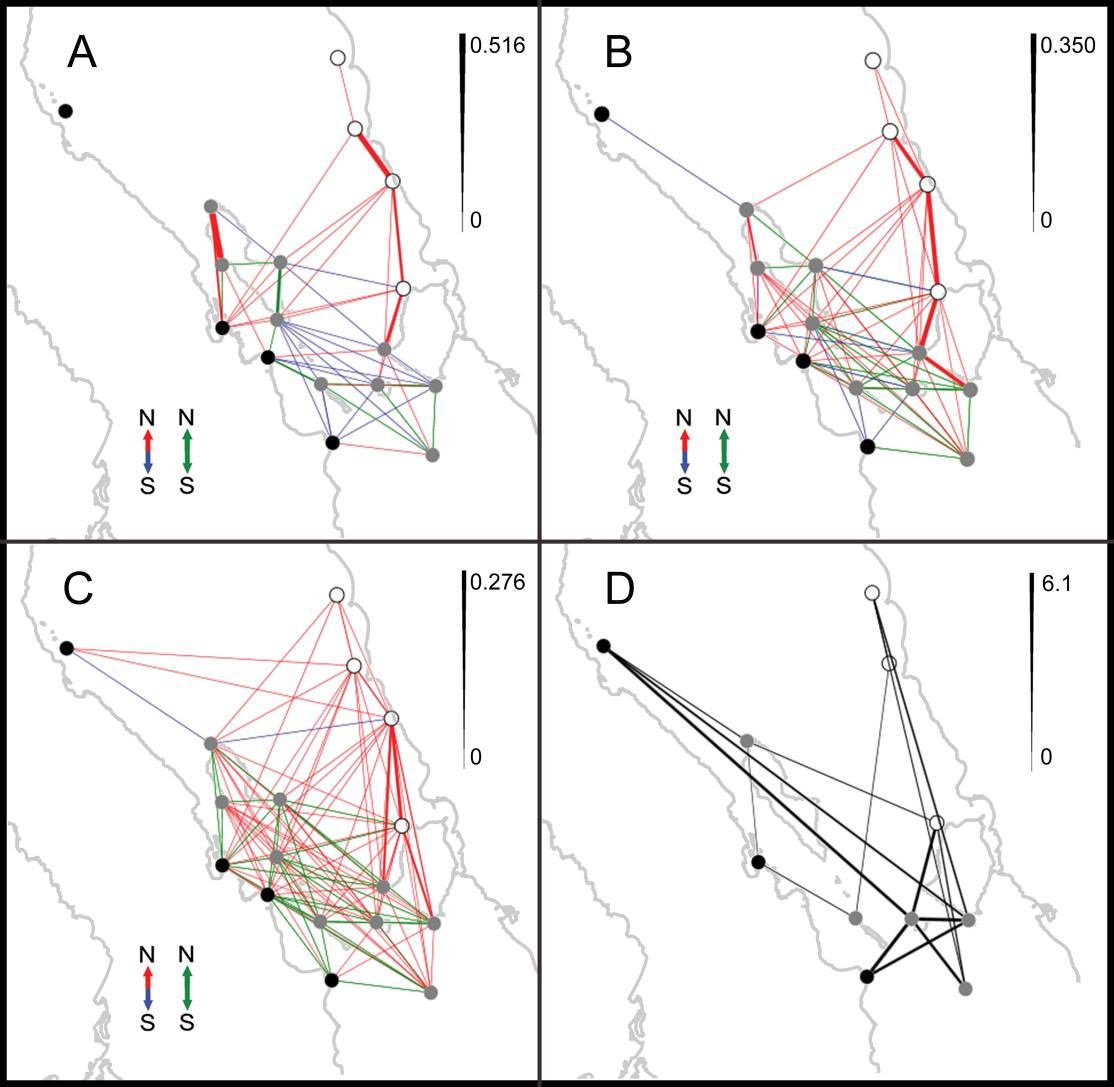
Modeled and empirical networks of larval connectivity. Spatial networks of larval dispersal between sites for PLD 14 days (A), 21 days (B) and 28 days (C), and conditional genetic distance from a population graph based on mtDNA sequence data (D), showing dispersal events (links) between sites (nodes). In (A)–(C) line width is proportional to probability, according to the scale to the right; the direction of the larval dispersal events is indicated by different colors: northward (red), southward (blue) or both simultaneously (green). The empirical network is undirected and line width is proportional to conditional genetic distance according to the scale. Black nodes belong to “Baja” geographic group, grey nodes belong to “Midriffs” and blank nodes to “Sonora”.

**Figure 5:**
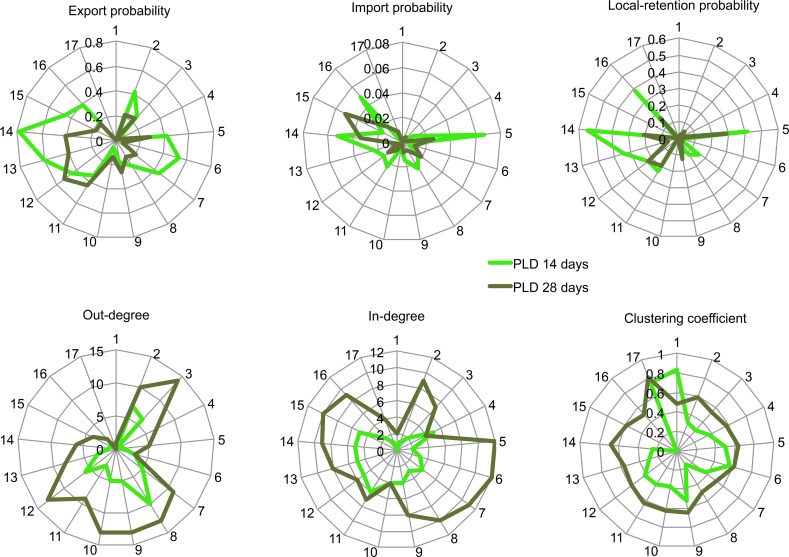
Performance of 17 sites for different aspects of marine connectivity. Performance was measured with export probability, import probability, local-retention probability, out-degree, in-degree and clustering coefficient, as estimated by an oceanographic model at PLD 14 and 28 days.

The trajectories of particles released from each site at the eight release dates (Video S1) showed sites in the Baja California Peninsula (sites 1–4) generally followed a southward direction in early May that gradually shifted to a northward direction during late May and June, while sites at the north edge of Tiburon Island (Site 14) and on mainland Sonora (sites 15–17) always followed a northward trajectory but the distance traveled by particles increased from May towards June. Most locations around the Midriff islands followed the same pattern described for sites 1–4, with the exception of San Pedro Martir Island and Datil island near the south end of Tiburon Island that had particles dispersing both north and south of the release site. In all cases, the distance traveled by particles was directly proportional to the PLD, which is not always true in such studies (e.g., Selkoe et al., 2010). The graph size of the connectivity networks increased from 57 edges at PLD 14 days, to 94 at PLD 21 days and 132 at PLD 28 days, indicating a longer PLD is associated with more connected and complex networks (Figs. 4A–4C). Based on the directionality of the links in the networks, three main patterns were evident. First, the proportion of southward links decreased from 22.8% at PLD 14, to 9.57% at PLD 21 and 1.51% at PLD 28; the proportion of northward and bi-directional (north–south) links was similar at all PLDs (ranging from 38.59% to 51.51%). Second, at PLD 14, northward links were prevalent along the sites located in the coast of Baja, in the Canal de Ballenas and between the southern end of Angel de la Guarda Island across the GC towards the northern end of Tiburon Island and mainland Sonora; southward links were present between the eastern and southern coasts of Angel de la Guarda Island towards southern locations in San Lorenzo Island and Baja California and across the Midriff Islands to Tiburon Island and San Esteban Island. Third, bi-directional links were evident in a small area located between San Pedro Martir Island, San Esteban and San Lorenzo Islands and the southern end of Tiburon Island at PLD 14; at PLD 21 they were found almost exclusively within sites in the Midriffs, while at PLD28 they were found at sites within Baja, within the Midriffs and between Baja and the Midriffs. In all PLDs, sites in Sonora received larvae from southern sites and were not involved in bi-directional links (Figs. 4A–4C). Overall, the strongest links (i.e., those showing the larger probabilities) were observed between the southern, western and northern coasts of Tiburon Island towards northern localities situated in mainland Sonora (Las Cuevitas and Puerto Libertad). Strong links were also present in the Canal de Ballenas at PLD 14 and PLD 21 (Figs. 5A and 5B).

The probability of local retention decreased with increasing PLD. According to the ocean model, local retention was most likely at the northern end of Angel de la Guarda Island (Punta Refugio, range: 0.41–0.28 for PLD 14 and 28 days, respectively), the northern end of Tiburon Island (El Tecomate, range: 0.54–0.21), followed by the southern coast of Tiburon Island (Datil Island, range: 0.23–0.19), San Esteban Island (range: 0.22–0.23) and Puerto Libertad (range: 0.38–0.16).

Our analyses illustrated that several of the connectivity metrics covary (including export, import and local retention probabilities), highlighting a few sites that were important despite variation in PLD (Fig. 5). For example, Puerto Refugio at the north of Angel de la Guarda Island and Tecomate on the north of Tiburon Island had among the largest probabilities of export, import and local retention. The sites with the largest probabilities of exporting larvae where located downstream, from site 11 (San Esteban Island) to 16 (Puerto Libertad), while those sites with the largest probabilities of importing larvae where located even more downstream matching the Sonora group (sites 14–16, with the exception of Puerto Refugio). In the graph theoretic analyses, out-degree peaked at the Baja group (sites 2 and 3) and within the Midriffs group (sites 7–12), while the Sonora group had overall low values. In contrast, in-degree was relatively high over the entire region, but particularly at downstream sites (11–16, Datil Island to Puerto Libertad). Clustering coefficient was highest at the most downstream site (Puerto Libertad) (Fig. 5). Betweenness centrality identified Las Cuevitas on mainland Sonora, the Southern end of Angel de la Guarda Island and La Ventana as key sites for multigenerational larval dispersal through the entire network (results not shown).

### Genetic connectivity

**Figure 6:**
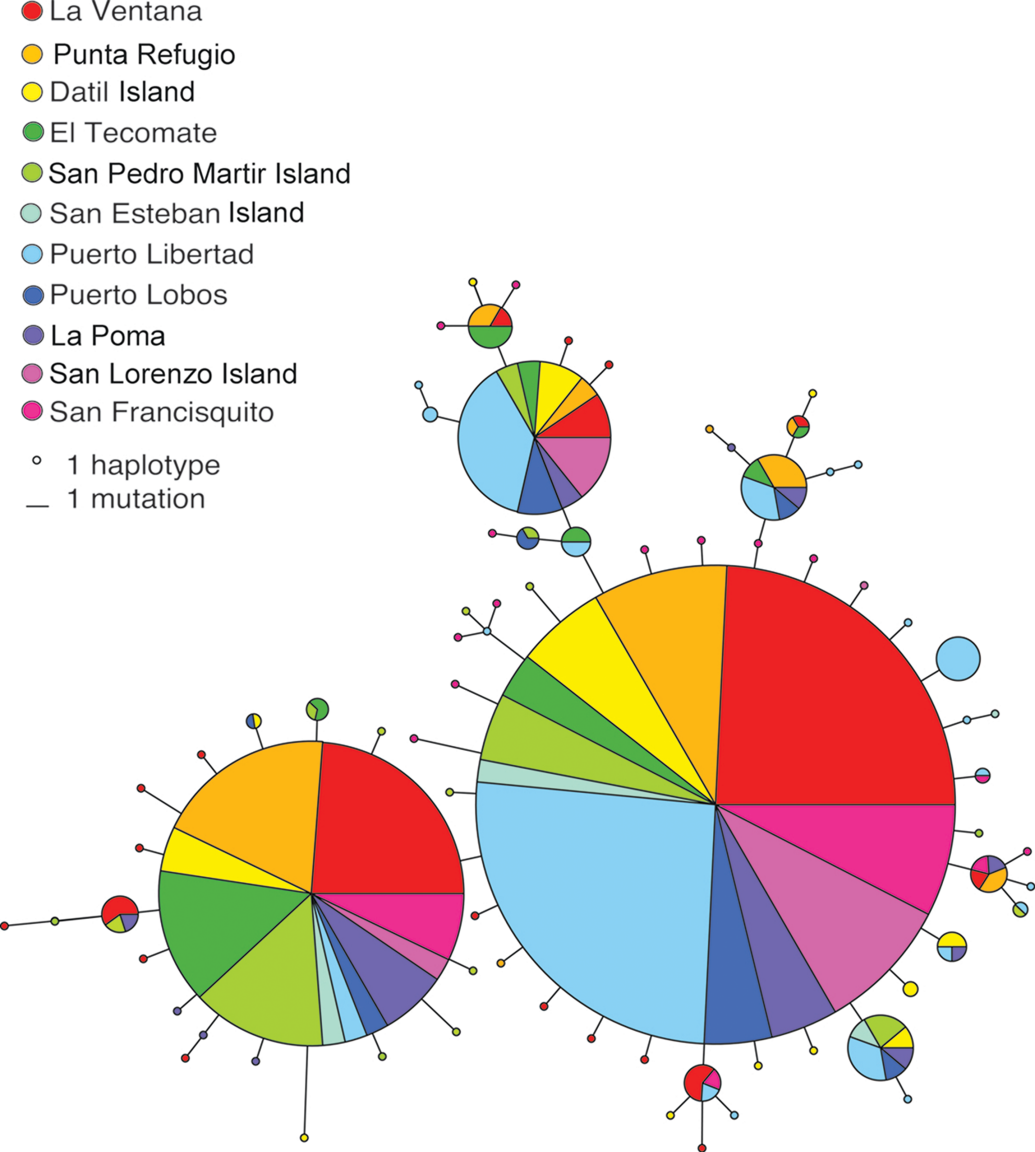
Minimum spanning network among haplotypes. The network shows the relationships among 79 haplotypes found in *M. rosacea*. Circles are sized proportionally to the number of individuals that possess each haplotype and colors indicate their geographic distribution in 11 sites shown to the left. All haplotypes are separated by one to four mutation steps as denoted by scaling provided.

We analyzed a 787 bp fragment of cytochrome *b* and a 726 bp fragment of ATPase for 260 individuals. We identified a total of 79 haplotypes (GenBank accesion numbers KJ004770–KJ004925), with adjacent haplotypes in the haplotype network separated by 1–4 bp (Fig. 6). There was limited evidence of geographic separation of haplotypes, and the three most frequent haplotypes were present in all locations, with the exception of the third most frequent which was absent in San Francisquito on the Baja Peninsula. Corrected estimates of haplotype diversity were high, ranging from 0.844 (San Lorenzo Island) to 0.943 (San Esteban Island) (Table 2). Nucleotide diversity was low, varying from 0.0011 (San Lorenzo Island) to 0.0020 (Datil Island).

Statistically significant pairwise estimates of genetic structure were observed (Table 3). Pairwise *F_ST_*, *ϕ_ST_*, and ${F}_{S T}^{{\prime}}$ values suggest Puerto Libertad is genetically divergent from the majority of other sampling localities, while La Poma and San Esteban Island are the most similar to all the other sites (Table 3, Table S1). Global estimates suggest moderate levels of population structure within the northern Gulf (*F_ST_* = 0.016, *P* = 0.011; *ϕ_ST_* = 0.0467, *P* = 0.00059; ${F}_{S T}^{{\prime}}=0.1413$). The *F_ST_*/*ϕ_ST_* ratio based on global values was 0.3426, indicating insufficient gene flow to homogenize populations and that closely related haplotypes show some geographical structure despite occasional long distance dispersal (Pons & Petit, 1996; Bird et al., 2011).

**Table 3:** Pairwise *F_ST_* and *ϕ_ST_* statistics between sites. Pairwise *F_ST_* values are above diagonal and pairwise *ϕ_ST_* values are below diagonal. For each site, we show their membership to one of three geographic groups. *P* values below 0.05 are shown in bold.

Group	Baja			Midriffs					Sonora		
Location	1	2	4	5	9	10	11	12	14	16	17
1. La Poma	–	−0.011	−0.018	−0.016	0.054	−0.017	−0.022	−0.090	−0.008	0.012	−0.023
2. La Ventana	−0.0124	–	−0.004	0.004	0.030	0.011	0.009	−0.054	0.025	**0.025**	0.004
4. San Francisquito	−0.0176	−0.0045	–	0.009	**0.057**	0.011	−0.005	−0.062	0.012	0.020	−0.001
5. Punta Refugio	−0.031	0.012	−0.002	–	**0.080**	−0.009	0.025	−0.052	0.003	**0.055**	0.017
9. San Lorenzo Island	0.0725	0.0381	0.0193	0.070	–	**0.098**	0.043	0.042	**0.118** ^*^	0.011	0.003
10. San Pedro Martir Island	−0.0196	−0.0122	−0.0055	0.015	0.0869	–	0.015	−0.097	0.000	**0.061**	0.004
11. Datil Island	−0.0169	−0.0001	−0.0129	0.042	−0.0090	−0.0018	–	−0.062	0.017	0.009	−0.022
12. San Esteban Island	−0.0578	−0.0421	−0.0455	0.017	0.0772	−0.0391	−0.0605	–	−0.041	−0.020	−0.069
14. El Tecomate	0.0038	0.0101	0.0092	0.008	0.0621	−0.0094	0.0169	0.0203	–	**0.060**	0.026
16. Puerto Libertad	**0.0891**	**0.0912**	**0.0601**	**0.091**	−0.0094	**0.1189** ^*^	**0.0408**	0.0386	**0.1234** ^*^	–	−0.015
17. Puerto Lobos	0.0554	**0.0567** ^*^	0.0193	0.054	−0.0353	0.0555	−0.0035	0.0193	0.0433	0.0122	–

**Notes.**

* Indicate statistical significance after correcting for multiple tests (critical *P* = 0.0009).

The population graph derived from the genetic covariance among sites included all 11 empirical sites and had a graph size with 17 edges (Fig. 4D). Despite its smaller size and complexity compared to the modeled networks, its topology resembled some of the links suggested by the models, particularly at PLD 28 days (e.g., the links between Puerto Refugio on the north of Angel de la Guarda Island and: La Poma and La Ventana on the Baja Peninsula, and El Tecomate on the north of Tiburon Island; the links between San Esteban Island and: El Tecomate, Datil Island, San Pedro Martir Island and San Francisquito on the Baja Peninsula).

**Figure 7:**
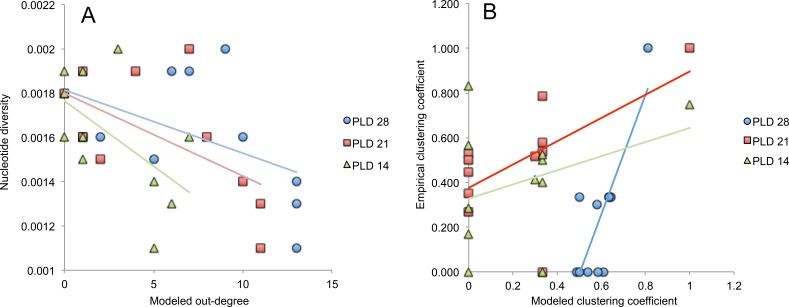
Node-base analyses. (A) Scatter plot between modeled estimates of out-degree at three PLDs and observed values of nucleotide diversity. (B) Scatter plot between modeled and empirical estimates of clustering coefficient.

Node-base analyses via fit of a linear model for explaining haplotype and nucleotide diversity using in-degree, out-degree and betweenness centrality at each PLD as explanatory variables revealed that nucleotide diversity was lower at locations showing higher out-degree according to the model at PLD 14 days (*P* = 0.0318, Table 4, Fig. 7A) and PLD 21 days (*P* = 0.0305), but patterns were not significant after correcting for multiple tests using FDR (critical *P* = 0.0029). No other comparisons were significant. The linear regression between modeled and empirical values of betweenness centrality and clustering coefficient at each PLD showed only the clustering coefficient estimates were correlated at PLD 21 days (*P* = 0.0487, *R*^2^ = 0.3655, Table 5, Fig. 7B) and PLD 28 days (*P* = 0.0019, *R*^2^ = 0.6729). After correcting for multiple tests, only patterns at PLD 28 days were significant (critical *P* = 0.0083).

**Table 4:** Node-based analyses for explaining genetic diversity. Results of fitting a linear model for explaining empirical corrected haplotype diversity and nucleotide diversity with modeled in-degree, out-degree and betweenness centrality as explanatory variables at three PLDs. For each variable we show the coefficient estimated in the model, its associated *P* value, the Incidence Risk Ratio (IRR) coefficient and the 95% lower (L) and upper (U) confidence intervals. For each model tested, we show the multiple *R*^2^ value and the associated *P* value. *P* values below 0.05 are shown in bold.

	Corrected haplotype diversity (*h*_†_)					Nucleotide diversity (*π*)				
	Coefficient	*P*-value	IRR	L	U	Coefficient	*P*-value	IRR	L	U
**PLD 14**										
In-degree	0.0048314	0.408	1.004843	0.9984597	1.011267	6.12E–05	0.1775	1.0000612	1.0000055	1.0001169
Out-degree	−0.0033951	0.458	0.9966107	0.9894915	1.003781	−8.61E–05	**0.0318**	0.9999139	0.99986	0.9999678
Betweenness	0.0002055	0.84	1.0002055	0.9988981	1.001515	9.74E–06	0.2229	1.0000097	0.9999954	1.0000241
Multiple *R*^2^	0.1445	0.7614				0.5147	0.1459			
**PLD 21**										
In-degree	0.0007668	0.871	1.0007671	0.9970901	1.0044577	4.51E–05	0.214	1.0000451	1.0000094	1.0000809
Out-degree	−0.0012069	0.616	0.9987938	0.994191	1.003418	−4.53E–05	**0.0305**	0.9999547	0.9999308	0.9999787
Betweenness	−0.0013393	0.23	0.9986616	0.9982158	0.9991075	−1.26E–05	0.1319	0.9999874	0.9999784	0.9999964
Multiple *R*^2^	0.2277	0.5875				0.5816	0.09045			
**PLD 28**										
In-degree	−0.002588	0.631	0.9974157	0.9895889	1.005305	5.04E–05	0.275508	1.0000504	0.9999974	1.0001033
Out-degree	−0.000758	0.71	0.9992423	0.9955642	1.002934	−3.23E–05	0.085887	0.9999677	0.9999465	0.9999889
Betweenness	−0.001097	0.726	0.998904	0.9947085	1.003117	−4.08E–05	0.144042	0.9999592	0.9999285	0.9999899
Multiple *R*^2^	0.23	0.5829				0.4607	0.2037			

**Notes.**

* Indicate statistical significance after correcting for multiple tests (critical *P* = 0.0029).

**Table 5:** Node-based analyses correlating modeled and empirical network metrics. Linear regression analyses between two node-base metrics (betweenness centrality and clustering coefficient) estimated from the modeled and empirical networks. *P* values below 0.05 are shown in bold.

PLD	Node-base metric	*R* ^2^	*P* value
PLD 14 days	Betweenness centrality	0.0060	0.8196
	Clustering coefficient	0.1201	0.2963
PLD 21 days	Betweenness centrality	0.0699	0.4319
	Clustering coefficient	0.3655	**0.0487**
PLD 28 days	Betweenness centrality	0.1189	0.2990
	Clustering coefficient	0.6729	**0.0019** ^*^

**Notes.**

* Indicate statistical significance after correcting for multiple tests (critical *P* = 0.0083).

**Figure 8:**
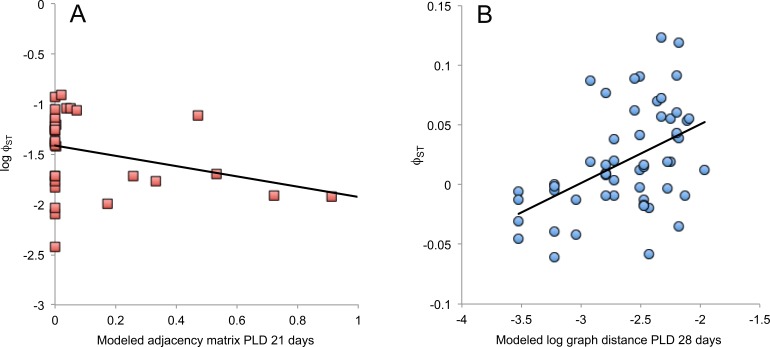
Matrix-based analyses. (A) Scatter plot between adjacency values from the modeled network at PLD 21 days and empirical log *ϕ_ST_* values. (B) Scatter plot between log graph distances estimated from the modeled network at PLD 28 days and empirical *ϕ_ST_* values.

Matrix-based analyses showed a lack of correlation between pairwise *ϕ_ST_*, *F_ST_* and ${F}_{S T}^{{\prime}}$ values against geographic distance (Mantel test *P* = 0.386, *R*^2^ = 0.0004, Table 6; *P* = 0.8080, *R*^2^ = 0.0262, Table S2; *P* = 0.6970, *R*^2^ = 0.0056, Table S3, respectively). In contrast, we found significant correlations supporting our predictions at all PLDs between the adjacency and graph distance matrices calculated from the modeled networks and the empirical log *ϕ_ST_* matrix, while controlling for geographic distance (Table 6). First, genetic differentiation decreased between sites with larger adjacency values indicative of high larval dispersal probability, particularly at PLD 21 days (partial Mantel test *P* < 0.0001, *R*^2^ = 0.1213, Fig. 8A). Second, genetic differentiation increased between sites with larger graph distance, especially at PLD 28 days (partial Mantel test *P* < 0.0001, *R*^2^ = 0.0684). The *ϕ_ST_* matrix and the (log) graph distance matrix were also significantly correlated at PLD 28 days (Mantel test *P* = 0.0060, *R*^2^ = 0.2224, Fig. 8B), further corroborating a significant trend of low genetic structure associated with nodes located nearby according to the topology of the modeled network of larval dispersal. Similar analyses employing *F_ST_* and ${F}_{S T}^{{\prime}}$ values showed significant patterns only when compared against adjacency values, particularly at PLD 14 days (*F_ST_P* < 0.0001, *R*^2^ = 0.1177, Table S2; ${F}_{S T}^{{\prime}}P\lt 0.0001$, *R*^2^ = 0.1423, Table S3). Additionally, while modeled and empirical graph distance matrices were not significantly correlated at any PLD according to linear regression analyses (All *P* values ≥ 0.1402, all *R*^2^ ≤ 0.0405, Table 7), the adjacency matrices were significantly correlated at PLD 21 days (Linear regression *P* = 0.0065, *R*^2^ = 0.1315).

**Table 6:** Matrix-based analyses for explaining patterns of genetic structure. Mantel and partial Mantel tests between an empirical matrix of genetic structure (*ϕ_ST_*) values and various explanatory variables, including geographic distance and three distinct matrices calculated from the modeled networks, including adjacency, graph distance and log graph distance. *P* values below 0.05 are shown in bold.

	Matrix 1	Matrix 2	Controlling matrix	*P* value	*R* ^2^
	*ϕ_ST_*	GeoD	–	0.386	0.0004
**PLD 14**	log *ϕ_ST_*	Adjacency	log GeoD	**<0.00001** ^*^	0.0660
	log *ϕ_ST_*	GraphD	log GeoD	**<0.00001** ^*^	0.0371
	*ϕ_ST_*	log GraphD	–	0.202	0.0306
**PLD 21**	log *ϕ_ST_*	Adjacency	log GeoD	**<0.0001** ^*^	0.1213
	log *ϕ_ST_*	GraphD	log GeoD	**<0.0001** ^*^	0.0241
	*ϕ_ST_*	log GraphD	–	0.29	0.0123
**PLD28**	log *ϕ_ST_*	Adjacency	log GeoD	**<0.0001** ^*^	0.0487
	log *ϕ_ST_*	GraphD	log GeoD	**<0.0001** ^*^	0.0684
	*ϕ_ST_*	log GraphD	–	**0.0060** ^*^	0.2224

**Notes.**

* Indicate statistical significance after correcting for multiple tests (critical *P* = 0.0060).

**Table 7:** Matrix-based analyses correlating empirical and modeled networks. Linear regression analyses between two types of matrices (adjacency and graph distance) estimated from the modeled and the empirical networks. *P* values below 0.05 are shown in bold.

PLD	Matrix	*R* ^2^	*P* value
PLD 14 days	Adjacency	0.0367	0.1608
	Graph distance	0.0116	0.4334
PLD 21 days	Adjacency	0.1315	**0.0065** ^*^
	Graph distance	0.0229	0.2693
PLD 28 days	Adjacency	0.0187	0.3193
	Graph distance	0.0405	0.1402

**Notes.**

* Indicate statistical significance after correcting for multiple tests (critical *P* = 0.0083).

Regional genetic subdivision was observed when sampling sites were clustered into the Baja Peninsula, Midriff Islands and Sonoran coast groups, as supported by a chi-squared test (*χ*^2^ = 186.876, d.f. = 152, *P* = 0.0286) and an AMOVA test (*F_ST_* = 0.0466, *P* < 0.05, Table S4). Rankings of proposed larval dispersal models between the aforementioned regions are listed in Table 8. The best-supported model of larval migration based on the genetic data was for unidirectional larval dispersal from the Baja Peninsula towards Sonora (File S1–S3). The analysis of modularity revealed the presence of two statistically supported sub-graphs in the modeled networks at all PLDs but with higher support at PLD 21 (Table 9, *P* = 0.0003). The first segment consisted of Baja + Midriffs and the second segment included Sonora. The empirical network did not show any evidence of modularity.

**Table 8:** Probability of three larval dispersal models based on the genetic data. Bayes factors and marginal log likelihoods estimated in Migrate-n version 3.2.16 using Bayesian approximation and thermal integration for three proposed larval dispersal models including: an unrestricted (Full matrix) larval dispersal among three groups (Baja, Midriffs and Sonora), a directional model of larval dispersal from Baja across the Midriffs towards Sonora (Baja to Sonora), and a directional model from Sonora across the Midriffs towards Baja (Sonora to Baja).

Model	Bezier l mL	Harmonic l mL	Choice (Bezier)	Model probability
Full matrix	−2673.20	−2514.33	2	0.00001
Baja to Sonora	−2658.64	−2513.07	1	0.99999
Sonora to Baja	−2974.33	−2529.21	3	0.00000

**Table 9:** Probability of sub-graph structure. *P*-values supporting the presence of sub-graph structure among three defined geographic groups (Baja, Midriffs and Sonora) for the modeled connectivity networks at PLD 14 days, PLD 21 days and PLD 28 days, and the empirical network from genetic data. Distinct segments of the proposed sub-graphs are separated by a “/” symbol, while a “+” symbol denotes groups considered as a single segment. *P* values below 0.05 are shown in bold.

Sub-graph	PLD 14 days	PLD 21 days	PLD 28 days	Empirical network
Baja / Midriffs + Sonora	0.4724	0.1732	0.3645	0.6064
Baja + Midriffs / Sonora	**0.0006** ^*^	**0.0003** ^*^	**0.0017** ^*^	0.4086
Midriffs / Baja + Sonora	0.1074	**0.0439**	0.1739	0.9940

**Notes.**

* Indicate statistical significance after correcting for multiple tests (critical *P* = 0.0041).

## Discussion

Our study contributes to a growing body of literature (Galindo et al., 2010; Selkoe et al., 2010; Alberto et al., 2011; Crandall, Treml & Barber, 2012; Foster et al., 2012; Petitgas et al., 2012; Soria et al., 2012; Feutry et al., 2013) highlighting the inherent value of verifying outputs of biophysical oceanographic models with empirical genetic data to inform larval dispersal patterns and marine connectivity. We corroborated our hypothesis that an oceanographic model describing metapopulation dynamics of larval dispersal in the GC can help to explain empirical genetic patterns. The model helped to explain genetic differences between sites but not genetic diversity within sites, which could result from the combination of using a single relatively slow evolving genetic marker (a protein-coding mitochondrial DNA marker) and the fact that genetic variation between sites evolves more slowly than within sites (Keyghobadi et al., 2005). We predict that the use of multiple fast-evolving and hypervariable markers (e.g., microsatellites), could improve the power of the model to predict genetic diversity within sites. Nevertheless, significant correlations between node-based modeled and empirical clustering coefficient suggest that summarizing genetic data with a population graph increased the power of the model to explain the empirical data. Validation of the passive dispersal model through subsequent studies such as those using parentage analyses and highly polymorphic microsatellite loci are recommended and underway.

Concordance of pairwise genetic differences and matrices describing networks derived from biophysical modeling data for *M. rosacea* elucidate the role of oceanographic processes in driving patterns of larval dispersal while models helped to explain seemingly chaotic patterns of genetic structure. Our study shows that in addition to contrasting pairwise values of genetic structure to modeled probabilities (Selkoe et al., 2010), or modeled oceanographic distances (White et al., 2010b; Alberto et al., 2011), graph distances over modeled networks or larval dispersal are a simple and promising tool to summarize marine connectivity patterns. The model using graph distances explained a larger variance in *ϕ_ST_* values than the model using adjacency matrices, indicating that although the presence or absence of a link and its probability are relevant, even more important are the topological order and relationships among sites driven by the prevailing oceanographic currents. For instance, Puerto Libertad on mainland Sonora showed the largest genetic differences compared to other sites, which could be explained by its extreme downstream position (i.e., large graph distance values with most sites), along with large levels of local retention according to the model (Fig. 5) that could have contributed to a high proportion of kin than expected by chance and to high levels of genetic differentiation (Iacchei et al., 2013).

Although the Baja, Midfriffs and Sonora groups were significantly differentiated, the best supported gene flow model (based on genetic subdivisions) agreed with the general cyclonic (anti-clockwise) direction of the gyre in the northern GC during the spawning period of *M. rosacea* that transports larvae from the Baja Peninsula, across the GC and towards the Sonoran coastline. This empirical result is in line with the analysis of graph modularity on the modeled networks that strongly suggested a larger bi-directional exchange of larvae between the Baja and the Midriffs group that were relatively separated from the Sonora group that was a net importer (sink) of larvae from upstream sites.

Our study had several limitations, with the major ones discussed below. While there is no direct evidence that leopard grouper larvae vertically migrate to escape advecting currents, there is a growing body of evidence that suggests that local retention of larvae in groupers can be quite high (Harrison et al., 2012; Almany et al., 2013). Thus our passive model could have overestimated larval exchange rates and underestimated local retention (Cowen et al., 2000; Largier, 2003; Leis, 2007). The effects of vertical migration could sometimes be comparable to those of reducing PLD (Andrello et al., 2013), or substantially increase or decrease dispersal (Woodson & McManus, 2007). Investigations into larval behavior of groupers are warranted and could greatly increase the precision and accuracy of the model. Our models did not include an explicit description of the habitat for larval recruitment, such as beads of the macro algae *Sargassum* sp. (Aburto-Oropeza et al., 2007), nor did they consider larval mortality after settlement. Thus, we only assessed potential connectivity, as opposed to realized connectivity (sensu Watson et al., 2010).

By coupling modeled and empirical connectivity approaches, we are able to better understand the mechanisms driving dispersal in the GC and inform spatially explicit management efforts for *M. rosacea* as well as marine organisms with similar life histories. Our multidisciplinary approach provided a mechanistic explanation of why some areas in the Midriff Island region concentrate the fishing effort for leopard grouper in the GC. Most of the largest and most heavily targeted fishing areas, including the north end of Angel de la Guarda Island, the west, north and south edges of Tiburon Island and Las Cuevitas and Puerto Libertad on mainland Sonora (Moreno-Baez et al., 2010; Moreno-Báez et al., 2012), showed the largest values of local retention of larvae, together with a high probability of importing larvae from other spawning sites and for concentrating larvae from all over the region. Notably, some of these sites are known to historically hold huge spawning aggregations of leopard grouper that have been harvested at high levels for decades, like the north end of Angel de la Guarda Island (Cannon, 1966). Thus, the main fishing areas seem to depend simultaneously on both local retention and contributions of larvae from upstream sites, coupled to oceanographic patterns that focus larval density towards these areas that sustain most of the fisheries.

A key result of our study is the observation that marine connectivity for *M. rosacea* from Baja California Peninsula and the Midriff island region towards Sonora is predominantly asymmetric. Other studies have previously shown the negative effects that asymmetric connectivity has on population persistence (Bode, Burrage & Possingham, 2008; Vuilleumier, Bolker & Leveque, 2010). In the presence of strong asymmetric currents, reserves (no-take zones) can significantly outperform traditional quota based management strategies in terms of fisheries yield, with considerably less risk (Gaines, Gaylord & Largier, 2003). Asymmetry also constrains the notion that benefits of reserves in terms of larval input are proportional to their distance to the reserve (Almany et al., 2009; Buston et al., 2012). For example, one study using DNA parentage analyses found that reserves in the Great Barrier Reef, which accounted for 28% of the local reef area, produced approximately half of all juvenile recruitment of snappers and groupers to both reserve and fished reefs within 30 km of the source spawning site inside the reserve (Harrison et al., 2012). A similar study in Papua New Guinea used parentage to track larval dispersal and predicted that 50% of larvae in a coral grouper settled within 13 km of the spawning aggregation sites (Almany et al., 2013). In contrast, the benefits of reserves in the Midriff Island region are completely biased towards one particular direction towards Sonora, highlighting that the spatial location of no-take zones to ensure connectivity is even more important than the number of zones, compared to other systems.

A network of no-take zones within the Midriff region might very well have a defined zone of influence that does not include the eastern edge of Tiburon Island or any locality towards the south in mainland Sonora. This observation has important practical implications. For example, fishing localities on mainland Sonora South of Tiburon Island are restricted from fishing at no-take areas within MPAs in the Midriffs, yet according to this and another study (Soria et al., 2014) localities south of Tiburon Island receive few benefits by not fishing there. Conversely, fishing communities in mainland Sonora (Puerto Lobos, Puerto Libertad) seem to receive great benefits from San Pedro Martir, San Esteban and Tiburon Islands, even though fishers from Puerto Lobos and Puerto Libertad may not fish there. These observations bring up an important concept in highly advective systems like the GC where there may be a spatial disconnect and strong directionality between the location of no-take zones and the areas that benefit most from them, and highlights that in order for such reserves to be effective they need to be located upstream of targeted fishing sites (Beger et al., 2010). Non-traditional approaches, such as “larval credits” based on regional larval export production (Kough, Paris & Butler, 2014) could help to manage such trade-offs. Our analyses suggest that establishment of smaller no-take zones at the north end of Angel de la Guarda Island within the current MPA will likely boost local fisheries via local retention, while other existing no-take zones within the Canal de Ballenas and San Lorenzo MPA could export larvae to fishing sites across the GC. The establishment of additional no-take zones adjacent to currently heavily fished areas in the western and northern edges of Tiburon Island, and in the coast between Las Cuevitas-Puerto Lobos will likely increase productivity of local fisheries (via local retention) and also fisheries at downstream fished sites on mainland Sonora as north as Puerto Peñasco (located ∼300 km from Tiburon Island) via larval dispersal. Notably, except for San Francisquito on the coast of Baja California, current MPAs do not include downstream sink sites receiving larvae from multiple sources which harbor the largest genetic diversity and evolutionary potential (San Esteban and Tiburon Islands). Our findings highlight that important upstream sites for improving fisheries are not necessarily aligned spatially with other criteria for protection, such as preserving evolutionary potential via genetic variation.

## Supplemental Information

10.7717/peerj.511/supp-1 Figure S1 Spatial units of analyses for studying connectivity in the Gulf of California The map shows 17 sites where virtual larvae were released (colored polygons) and 11 sites where genetic samples were also collected (blue polygons).

10.7717/peerj.511/supp-2 Figure S2 Tidal range during May and June in the Gulf of California Tides in the HAMSOM oceanographic model for the northern Gulf of California, showing the four neap and four spring tides during May and June selected for the release of virtual larvae. Red lines separate distinct months.

10.7717/peerj.511/supp-3Table S1Pairwise maximum *F_ST_* possible and ${F}_{S T}^{{\prime}}$ statistics between sitesPairwise maximum *F_ST_* possible while maintaining the within-population diversity above diagonal and pairwise ${F}_{S T}^{{\prime}}$ values below diagonal. For each site, we show their membership to one of three geographic groups.

10.7717/peerj.511/supp-4 Table S2 Matrix-based analyses for explaining patterns of genetic structure (*F_ST_*) Mantel and partial Mantel tests between an empirical matrix of genetic structure (*F_ST_*) values and various explanatory variables, including geographic distance and three distinct matrices calculated from the modeled networks, including adjacency, graph distance and log graph distance. *P* values below 0.05 are shown in bold. * Indicate statistical significance after correcting for multiple tests (critical *P* = 0.0060).

10.7717/peerj.511/supp-5Table S3Matrix-based analyses for explaining patterns of genetic structure (${F}_{S T}^{{\prime}}$)Mantel and partial Mantel tests between an empirical matrix of genetic structure (${F}_{S T}^{{\prime}}$) values and various explanatory variables, including geographic distance and three distinct matrices calculated from the modeled networks, including adjacency, graph distance and log graph distance. *P* values below 0.05 are shown in bold. * Indicate statistical significance after correcting for multiple tests (critical *P* = 0.0060).

10.7717/peerj.511/supp-6 Table S4 Analysis of molecular variance (AMOVA) Analysis of molecular variance testing for regional genetic subdivision when sampling sites were clustered into the Baja Peninsula, Midriff Islands and Sonoran coast groups.

10.7717/peerj.511/supp-7 Video S1 Larval dispersal from each spawning site at eight release dates Maps showing the spatial distribution of larvae released at each of eight release dates corresponding to four neap and four spring tides during May and June at each of 17 sites (“X” in each box) for PLD 14, 21 and 28 days. Scale in kilometers.

10.7717/peerj.511/supp-8 File S1 Results from Migrate-n for unrestricted gene flow Detailed results from three independent runs with Migrate-n software for unrestricted gene flow among Baja, the Midriff Islands and Sonora.

10.7717/peerj.511/supp-9 File S2 Results from Migrate-n software for gene flow towards Sonora Detailed results from three independent runs with Migrate-n software for a model of gene flow from Baja across the Midriff Islands towards Sonora.

10.7717/peerj.511/supp-10 File S3 Results from Migrate-n software for gene flow towards Baja Detailed results from three independent runs with Migrate-n software for a model of gene flow from Sonora across the Midriff Islands towards Baja.

## Additional Information and Declarations

### Field Study Permissions

The following information was supplied relating to field study approvals (i.e., approving body and any reference numbers): Scientific collection permits were acquired from SAGARPA (No. DGOPA.09151.260809.2885 and DAPA/2/020511/01197).

### DNA Deposition

The following information was supplied regarding the deposition of DNA sequences: GenBank: KJ004770–KJ004925.
