# Supplementary figures and images for: Asymmetric connectivity of spawning aggregations of a commercially important marine fish using a multidisciplinary approach
Source: PeerJ. 2014 Aug 7;2:e511. doi: 10.7717/peerj.511 (PMC4137664; doi:10.7717/peerj.511)

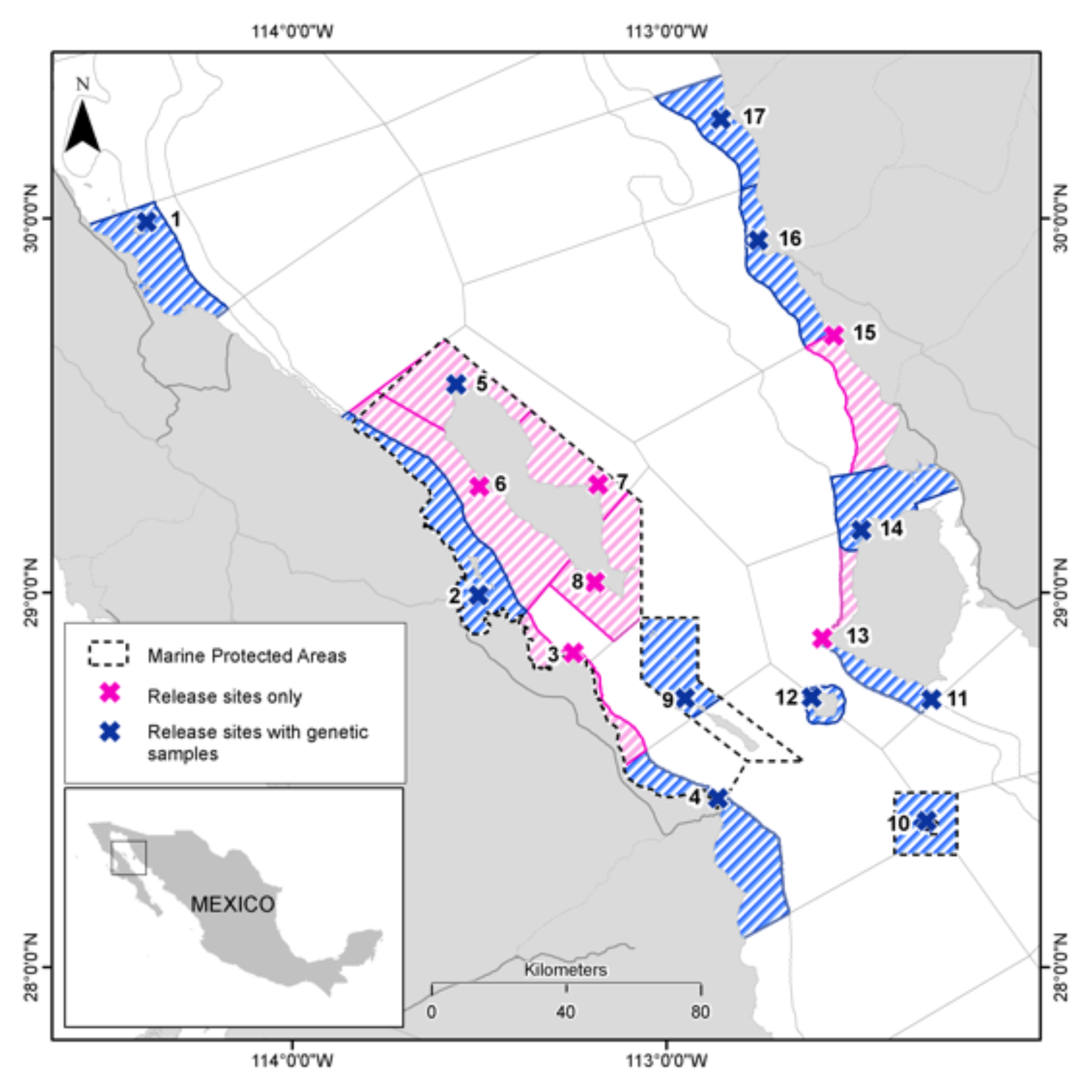

Supplement: Figure S1 — The map shows 17 sites where virtual larvae were released (colored polygons) and 11 sites where genetic samples were also collected (blue polygons). [file peerj-02-511-s001.png]

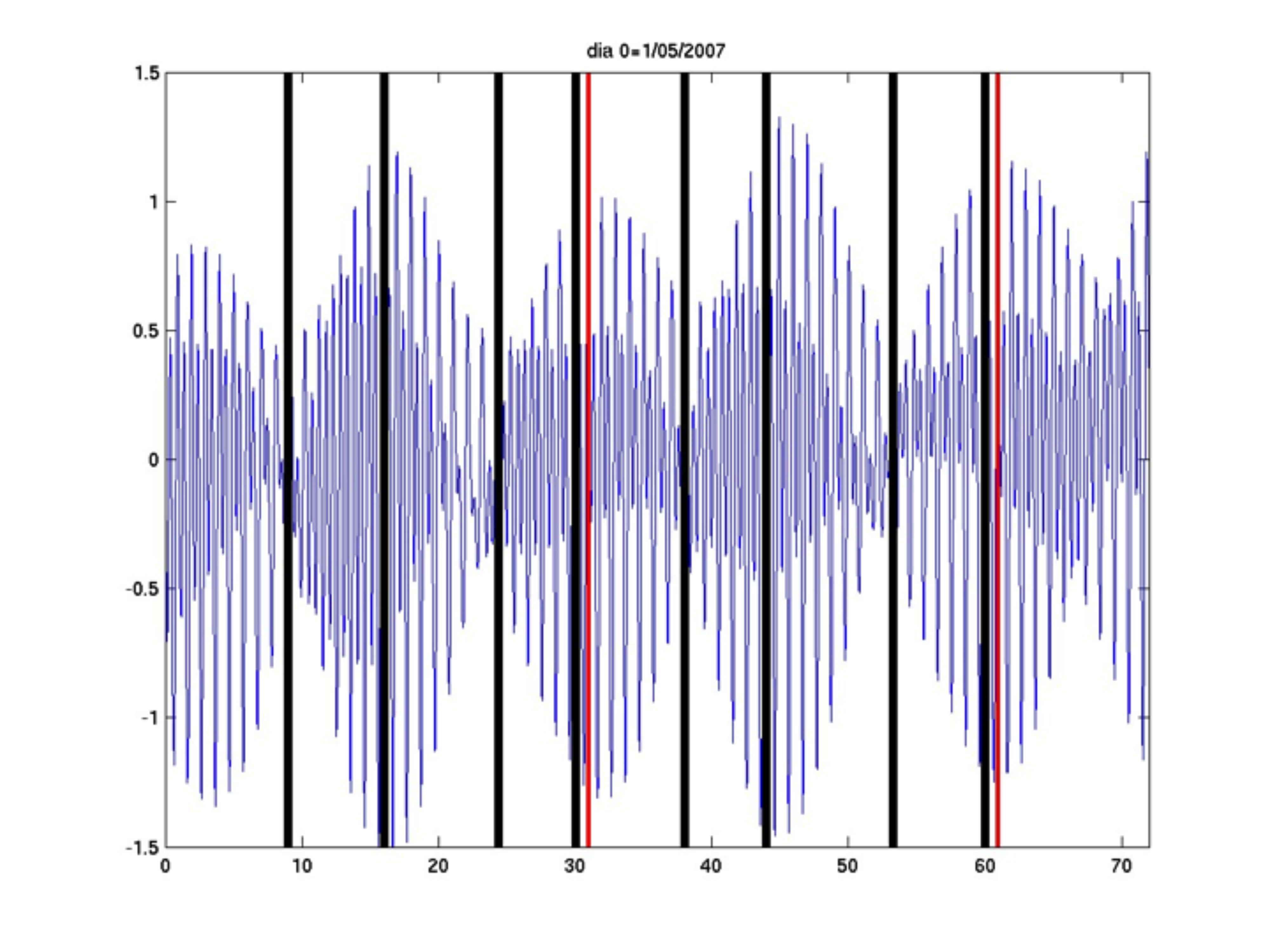

Supplement: Figure S2 — Tides in the HAMSOM oceanographic model for the northern Gulf of California, showing the four neap and four spring tides during May and June selected for the release of virtual larvae. Red lines separate distinct months. [file peerj-02-511-s002.png]
