# Supplementary material for: Asymmetric connectivity of spawning aggregations of a commercially important marine fish using a multidisciplinary approach
Source: PeerJ. 2014 Aug 7;2:e511. doi: 10.7717/peerj.511 (PMC4137664; doi:10.7717/peerj.511)
Supplement: Table S1 — Pairwise maximum FST possible while maintaining the within-population diversity above diagonal and pairwise ${F}_{S T}^{{\prime}}$FST′ values below diagonal. For each site, we show their membership to one of three geographic groups. [file peerj-02-511-s003.docx]

Table S1

| Group | Baja | | | Midriffs | | | | | Sonora | | |
| --- | --- | --- | --- | --- | --- | --- | --- | --- | --- | --- | --- |
| Location | 1 | 2 | 4 | 5 | 9 | 10 | 11 | 12 | 14 | 16 | 17 |
| 1. La Poma  2. La Ventana | -  -0.1183 | 0.0935  - | 0.0507  0.0929 | 0.108  0.144 | 0.1803  0.2089 | 0.0951  0.1342 | 0.0513  0.0939 | 0.0915  0.0837 | 0.0576  0.0988 | 0.0915  0.1278 | 0.0696  0.1130 |
| 4. San Francisquito | -0.3529 | -0.0430 | - | 0.106 | 0.1747 | 0.0943 | 0.0520 | 0.0317 | 0.0581 | 0.0910 | 0.0697 |
| 5. Punta Refugio | -0.1495 | 0.0278 | 0.0849 | - | 0.232 | 0.150 | 0.114 | 0.102 | 0.105 | 0.142 | 0.129 |
| 9. San Lorenzo Island | 0.3000 | 0.1435 | 0.3257 | 0.3448 | - | 0.2262 | 0.1776 | 0.2039 | 0.1800 | 0.2064 | 0.2091 |
| 10. San Pedro Martir Island | -0.1789 | 0.0821 | 0.1170 | -0.0600 | 0.4336 | - | 0.0955 | 0.0868 | 0.1006 | 0.1321 | 0.1167 |
| 11. Datil Island | -0.4314 | 0.0957 | -0.0962 | 0.2336 | 0.2416 | 0.1579 | - | 0.0323 | 0.0587 | 0.0920 | 0.0706 |
| 12. San Esteban Island | -3.0000 | -0.6429 | -1.9375 | -0.5098 | 0.2059 | -1.1149 | -1.9375 | - | 0.0401 | 0.0812 | 0.0542 |
| 14. El Tecomate | -0.1379 | 0.2525 | 0.2069 | 0.0268 | 0.6556 | 0.0000 | 0.2881 | -1.0250 | - | 0.0969 | 0.0767 |
| 16. Puerto Libertad | 0.1319 | 0.1953 | 0.2198 | 0.3873 | 0.0534 | 0.4621 | 0.0978 | -0.2469 | 0.6186 | - | 0.1109 |
| 17. Puerto Lobos | -0.3286 | 0.0354 | -0.0143 | 0.1318 | 0.0144 | 0.0342 | -0.3099 | -1.2778 | 0.3377 | -0.1351 | - |
