# Supplementary material for: Asymmetric connectivity of spawning aggregations of a commercially important marine fish using a multidisciplinary approach
Source: PeerJ. 2014 Aug 7;2:e511. doi: 10.7717/peerj.511 (PMC4137664; doi:10.7717/peerj.511)
Supplement: Table S2 — Mantel and partial Mantel tests between an empirical matrix of genetic structure (FST) values and various explanatory variables, including geographic distance and three distinct matrices calculated from the modeled networks, including adjacency, graph distance and log graph distance. P values below 0.05 are shown in bold. * Indicate statistical significance after correcting for multiple tests (critical P = 0.0060). [file peerj-02-511-s004.docx]

Table S2

|  | **Matrix 1** | **Matrix 2** | **Controlling**  **matrix** | **P value** | **R^2^** |
| --- | --- | --- | --- | --- | --- |
|  | *F_ST_* | GeoD | - | 0.8080 | 0.0262 |
| **PLD 14** | log *F_ST_* | Adjacency | log GeoD | **<0.0001*** | 0.1177 |
|  | log *F­_ST_* | GraphD | log GeoD | 0.9356 | 0.0198 |
|  | *F­_ST_* | log GraphD | - | 0.7000 | 0.0158 |
| **PLD 21** | log *F­_ST_* | Adjacency | log GeoD | **<0.0001*** | 0.0846 |
|  | log *F­_ST_* | GraphD | log GeoD | 0.9760 | 0.0565 |
|  | *F­_ST_* | log GraphD | - | 0.6640 | 0.0081 |
| **PLD28** | log *F­_ST_* | Adjacency | log GeoD | **<0.0001*** | 0.0044 |
|  | log *F­_ST_* | GraphD | log GeoD | 0.9995 | 0.0183 |
|  | *F­_ST_* | log GraphD | - | 0.2500 | 0.0246 |
