# Supplementary material for: Asymmetric connectivity of spawning aggregations of a commercially important marine fish using a multidisciplinary approach
Source: PeerJ. 2014 Aug 7;2:e511. doi: 10.7717/peerj.511 (PMC4137664; doi:10.7717/peerj.511)
Supplement: Table S3 — Mantel and partial Mantel tests between an empirical matrix of genetic structure (${F}_{S T}^{{\prime}}$FST′) values and various explanatory variables, including geographic distance and three distinct matrices calculated from the modeled networks, including adjacency, graph distance and log graph distance. P values below 0.05 are shown in bold. * Indicate statistical significance after correcting for multiple tests (critical P = 0.0060). [file peerj-02-511-s005.docx]

Table S3

|  | **Matrix 1** | **Matrix 2** | **Controlling**  **matrix** | **P value** | **R^2^** |
| --- | --- | --- | --- | --- | --- |
|  | *F'_ST_* | GeoD | - | 0.6970 | 0.0056 |
| **PLD 14** | log *F'_­ST_* | Adjacency | log GeoD | **<0.0001*** | 0.1423 |
|  | log *F­'_ST_* | GraphD | log GeoD | 0.998 | 0.0001 |
|  | *F­'_ST_* | log GraphD | - | 0.5900 | 0.0001 |
| **PLD 21** | log *F­'_ST_* | Adjacency | log GeoD | **<0.0001*** | 0.1019 |
|  | log *F­'_ST_* | GraphD | log GeoD | 0.871 | 0.0739 |
|  | *F­'_ST_* | log GraphD | - | 0.6100 | 0.0010 |
| **PLD28** | log *F­'_ST_* | Adjacency | log GeoD | **<0.0001*** | 0.0026 |
|  | log *F­'_ST_* | GraphD | log GeoD | 0.9910 | 0.0029 |
|  | *F­'_ST_* | log GraphD | - | 0.1840 | 0.0376 |
