# Supplementary material for: Asymmetric connectivity of spawning aggregations of a commercially important marine fish using a multidisciplinary approach
Source: PeerJ. 2014 Aug 7;2:e511. doi: 10.7717/peerj.511 (PMC4137664; doi:10.7717/peerj.511)
Supplement: Table S4 — Analysis of molecular variance testing for regional genetic subdivision when sampling sites were clustered into the Baja Peninsula, Midriff Islands and Sonoran coast groups. [file peerj-02-511-s006.docx]

Table S4

| **Source of variation** | **degrees of freedom** | **Sum of squares** | **Variance components** | **Percentage of variation** | **Fixation indices** |
| --- | --- | --- | --- | --- | --- |
| Among groups | 2 | 12.084 | 0.05438 | 4.16 | *F_SC_* = 0.00526 |
| Among populations within groups | 8 | 11.000 | 0.00659 | 0.50 | *F_ST_* = 0.04668  *F_CT_* = 0.04163 |
| Within populations | 129 | 310.066 | 1.24524 | 95.33 |  |
| Total | 259 | 333.150 | 1.30621 |  |  |
