# Supplementary material for: Asymmetric connectivity of spawning aggregations of a commercially important marine fish using a multidisciplinary approach
Source: PeerJ. 2014 Aug 7;2:e511. doi: 10.7717/peerj.511 (PMC4137664; doi:10.7717/peerj.511)
Supplement: File S1 — Detailed results from three independent runs with Migrate-n software for unrestricted gene flow among Baja, the Midriff Islands and Sonora. [file peerj-02-511-s008.pdf]

Example: sequence data set wit two loci [simula

MIGRATION RATE AND POPULATION SIZE ESTIMATION
using the coalescent and maximum likelihood or Bayesian inference
Migrate-n version 3.2.6 [1776]
Program started at Thu Jan 31 09:22:07 2013
Program finished at Fri Feb 1 03:31:42 2013

Options

Datatype: DNA sequence data

Inheritance scalers in use for Thetas: 1.00
[Each Theta uses the (true) ineritance scalar of the first locus as a reference]
Random number seed: (with internal timer) 940661911
Start parameters:

Theta values were generated from the FST-calculation

M values were generated from the FST-calculation

Connection type matrix:
where m = average (average over a group of Thetas or M,
s = symmetric M, S = symmetric 4Nm, 0 = zero, and not estimated,
* = free to vary, Thetas are on diagonal

Population	1	2	3
1 BAJA	*	*	*
2 MIDR	*	*	*
3 MAIN	*	*	*

Order of parameters:

1	Θ_1	<displayed>
2	Θ_2	<displayed>
3	Θ_3	<displayed>
4	$M_{2->1}$	<displayed>
5	$M_{3->1}$	<displayed>
6	$M_{1->2}$	<displayed>

7	$M_{3->2}$	<displayed>
8	$M_{1->3}$	<displayed>
9	$M_{2->3}$	<displayed>

Mutation rate among loci: Mutation rate is constant

Analysis strategy: Bayesian inference

Proposal distributions for parameter

Parameter	Proposal
Theta	Slice sampling
M	Slice sampling

Prior distribution for parameter

Parameter	Prior	Minimum	Mean*	Maximum	Delta	Bins
Theta	Uniform	0.000000	50.000000	100.000000	10.000000	1500
M	Uniform	0.000000	37500.000000	75000.000000	7500.000000	1500

Markov chain settings:	Long chain
Number of chains	1
Recorded steps [a]	50000
Increment (record every x step [b]	100
Number of concurrent chains (replicates) [c]	1
Visited (sampled) parameter values [a*b*c]	5000000
Number of discard trees per chain (burn-in)	2500000

Multiple Markov chains:

Static heating scheme 20 chains with temperatures

5 2.40 2.10 2.00 1.95 1.80 1.65 1.50 1.40 1.25 1.10 1.00

Swapping interval is 1

Print options:	
Data file:	MRO_NGulf_direc.seq
Output file:	MRO_NGulf_subset_run1
Posterior distribution raw histogram file:	bayesfile
Print data:	No
Print genealogies [only some for some data type]:	None

Data summary

Datatype:	Sequence data
Number of loci:	1

Population	Locus	Gene copies
1 BAJA	1	30
2 MIDR	1	30
3 MAIN	1	30
Total of all populations	1	90

Bayesian Analysis: Posterior distribution table

Locus	Parameter	2.5%	25.0%	Mode	75.0%	97.5%	Median	Mean
1	Θ_1	0.00000	0.00000	0.03333	13.33333	72.93333	46.83333	48.74249
1	Θ_2	0.00000	0.00000	0.03333	0.53333	1.40000	0.56667	0.10249
1	Θ_3	1.86667	22.00000	27.10000	32.20000	95.40000	49.83333	49.94295
1	$M_{2->1}$	38700.0	44850.0	47125.0	51000.0	75000.0	53375.0	51785.6
1	$M_{3->1}$	35650.0	45800.0	53875.0	56700.0	69950.0	51225.0	48963.4
1	$M_{1->2}$	12600.0	24050.0	27975.0	30500.0	40100.0	25725.0	24033.5
1	$M_{3->2}$	7250.0	23150.0	27325.0	30100.0	32000.0	24375.0	22001.7
1	$M_{1->3}$	37550.0	51800.0	56475.0	62050.0	75000.0	56775.0	54603.6
1	$M_{2->3}$	39000.0	57850.0	64525.0	69000.0	75000.0	61275.0	58195.6

Bayesian Analysis: Posterior distribution over all loci

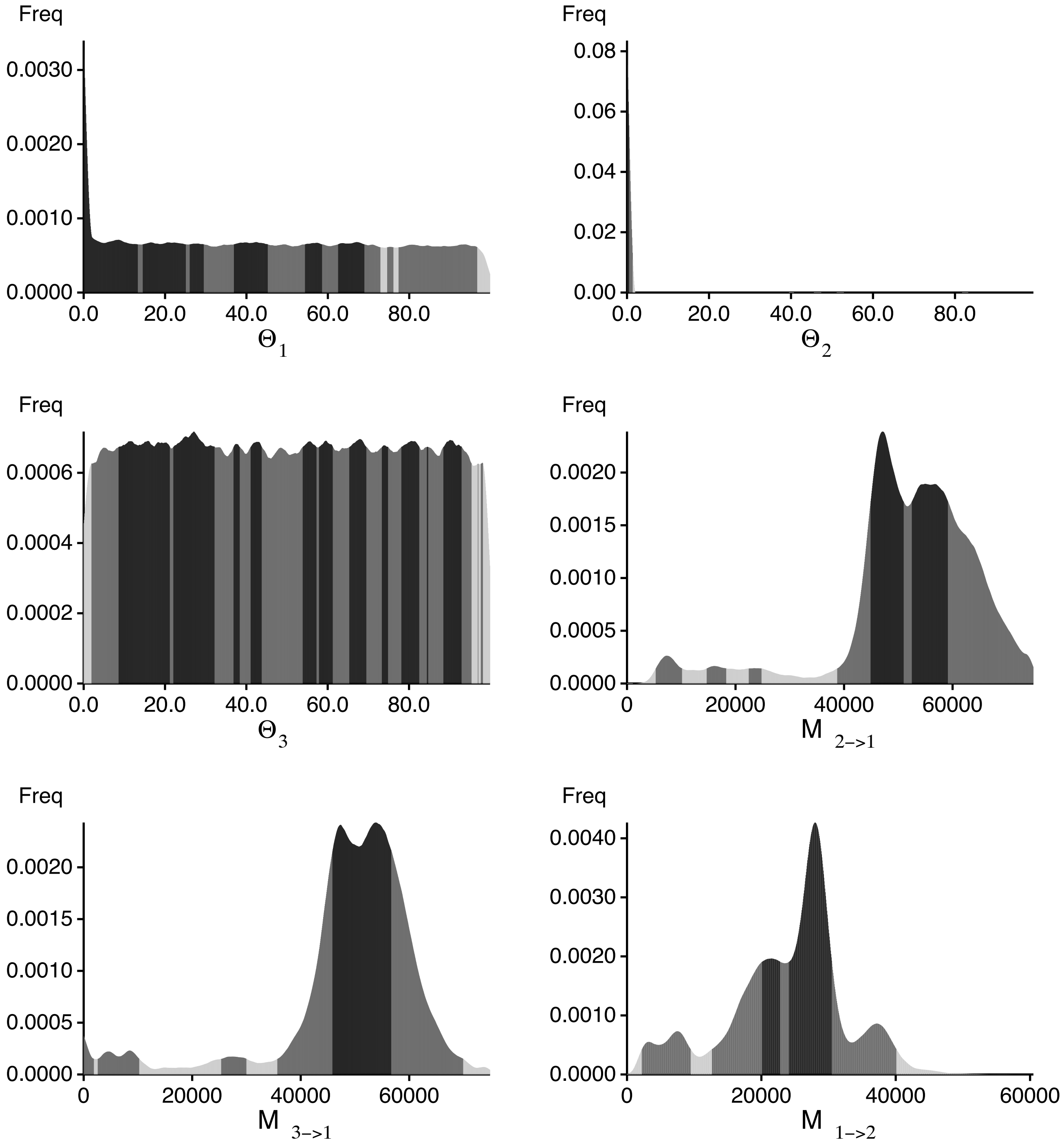

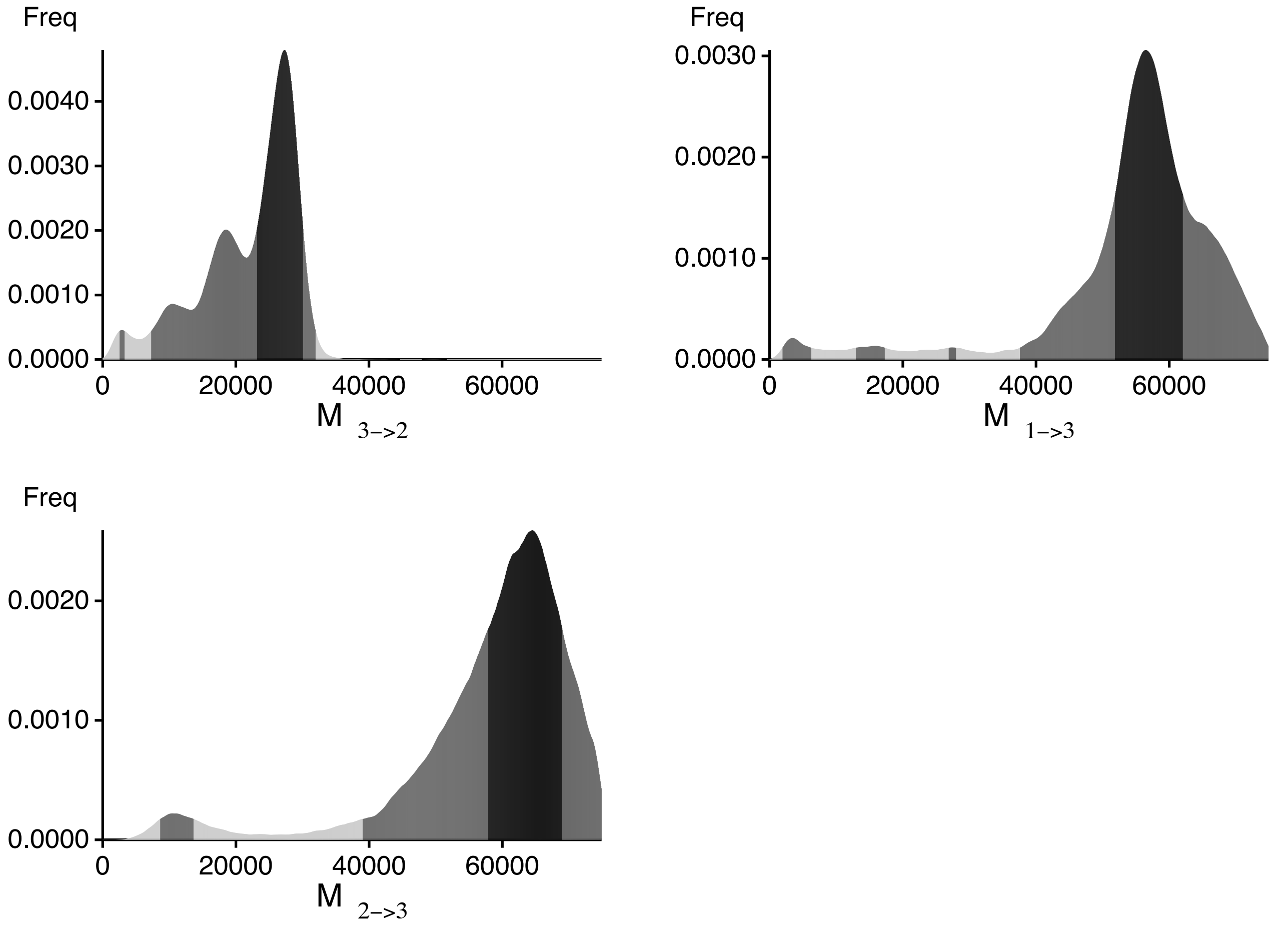
Freq
0.0040
0.0030
0.0020
0.0010
0.0000
0
20000
40000
60000
M 3->2
Freq
0.0030
0.0020
0.0010
0.0000
0
20000
40000
60000
M 1->3
Freq
0.0020
0.0010
0.0000
0
20000
40000
60000
M 2->3

Log-Probability of the data given the model (marginal likelihood)

Use this value for Bayes factor calculations:
BF = Exp[ln(Prob(D I thisModel) - ln(Prob(D I otherModel)
or as LBF = 2 (ln(Prob(D I thisModel) - ln(Prob(D I otherModel))
shows the support for thisModel]

Method	ln(Prob(DIModel))	Notes
Thermodynamic integration	-2673.945007	(1a)
	-2673.201840	(1b)
Harmonic mean	-2514.327699	(2)

(1a, 1b and 2) is an approximation to the marginal likelihood, make sure the program run long enough!
(1a, 1b) and (2) should give a similar result, (2) is considered more
crude than (1), but (1) needs heating with several well-spaced chains,
(1b) is using a Bezier-curve to get better approximations for runs with low number
of heated chains

Acceptance ratios for all parameters and the genealogies

Parameter	Accepted changes	Ratio
Θ_1	417266/417266	1.00000
Θ_2	416414/416414	1.00000
Θ_3	416618/416618	1.00000
$M_{2->1}$	417300/417300	1.00000
$M_{3->1}$	417026/417026	1.00000
$M_{1->2}$	415846/415846	1.00000
$M_{3->2}$	416676/416676	1.00000
$M_{1->3}$	416627/416627	1.00000
$M_{2->3}$	416463/416463	1.00000
Genealogies	269581/2500493	0.10781

MCMC-Autocorrelation and Effective MCMC Sample Size

Parameter	Autocorrelation	Effective Sampe Size
Θ_1	0.03325	46781.90
Θ_2	0.75541	6966.74
Θ_3	0.00608	49395.76
$M_{2->1}$	0.88258	3118.69
$M_{3->1}$	0.85248	3981.64
$M_{1->2}$	0.87851	3233.77
$M_{3->2}$	0.92880	1845.61
$M_{1->3}$	0.89045	2897.43
$M_{2->3}$	0.88126	3155.82
Ln[Prob(DIG)]	0.56377	13947.82

Warnings

You should most likely rerun your analysis after improving run parameters.

Param 9 (Locus 1): Upper prior boundary seems too low!

Example: sequence data set wit two loci [simula

MIGRATION RATE AND POPULATION SIZE ESTIMATION
using the coalescent and maximum likelihood or Bayesian inference
Migrate-n version 3.2.6 [1776]
Program started at Fri Feb 1 09:03:25 2013
Program finished at Sat Feb 2 03:12:08 2013

Options

Datatype: DNA sequence data

Inheritance scalers in use for Thetas: 1.00
[Each Theta uses the (true) ineritance scalar of the first locus as a reference]
Random number seed: (with internal timer) 1457695717
Start parameters:

Theta values were generated from the FST-calculation

M values were generated from the FST-calculation

Connection type matrix:
where m = average (average over a group of Thetas or M,
s = symmetric M, S = symmetric 4Nm, 0 = zero, and not estimated,
* = free to vary, Thetas are on diagonal

Population	1	2	3
1 BAJA	*	*	*
2 MIDR	*	*	*
3 MAIN	*	*	*

Order of parameters:

1	Θ_1	<displayed>
2	Θ_2	<displayed>
3	Θ_3	<displayed>
4	$M_{2->1}$	<displayed>
5	$M_{3->1}$	<displayed>
6	$M_{1->2}$	<displayed>

7	$M_{3->2}$	<displayed>
8	$M_{1->3}$	<displayed>
9	$M_{2->3}$	<displayed>

Mutation rate among loci: Mutation rate is constant

Analysis strategy: Bayesian inference

Proposal distributions for parameter

Parameter	Proposal
Theta	Slice sampling
M	Slice sampling

Prior distribution for parameter

Parameter	Prior	Minimum	Mean*	Maximum	Delta	Bins
Theta	Uniform	0.000000	50.000000	100.000000	10.000000	1500
M	Uniform	0.000000	37500.000000	75000.000000	7500.000000	1500

Markov chain settings:	Long chain
Number of chains	1
Recorded steps [a]	50000
Increment (record every x step [b]	100
Number of concurrent chains (replicates) [c]	1
Visited (sampled) parameter values [a*b*c]	5000000
Number of discard trees per chain (burn-in)	2500000

Multiple Markov chains:

Static heating scheme 20 chains with temperatures

5 2.40 2.10 2.00 1.95 1.80 1.65 1.50 1.40 1.25 1.10 1.00

Swapping interval is 1

Print options:	
Data file:	MRO_NGulf_direc.seq
Output file:	MRO_NGulf_subset_run2
Posterior distribution raw histogram file:	bayesfile
Print data:	No
Print genealogies [only some for some data type]:	None

Data summary

Datatype:	Sequence data
Number of loci:	1

Population	Locus	Gene copies
1 BAJA	1	30
2 MIDR	1	30
3 MAIN	1	30
Total of all populations	1	90

Bayesian Analysis: Posterior distribution table

Locus	Parameter	2.5%	25.0%	Mode	75.0%	97.5%	Median	Mean
1	Θ_1	0.80000	16.66667	19.10000	21.73333	26.53333	49.36666	49.77052
1	Θ_2	0.00000	0.00000	0.03333	0.53333	1.40000	0.56667	0.24806
1	Θ_3	0.00000	0.00000	0.03333	3.66667	22.93333	47.10000	49.47357
1	$M_{2->1}$	35300.0	46450.0	51075.0	58350.0	74550.0	52775.0	51771.4
1	$M_{3->1}$	36550.0	52550.0	59675.0	64700.0	75000.0	57625.0	54928.1
1	$M_{1->2}$	8400.0	16250.0	20075.0	22850.0	29400.0	19075.0	18828.0
1	$M_{3->2}$	10250.0	16600.0	19525.0	23550.0	34900.0	21675.0	21839.1
1	$M_{1->3}$	38050.0	53900.0	62175.0	66000.0	75000.0	58125.0	55651.6
1	$M_{2->3}$	40200.0	54250.0	59225.0	64500.0	75000.0	58975.0	56624.1

Bayesian Analysis: Posterior distribution over all loci

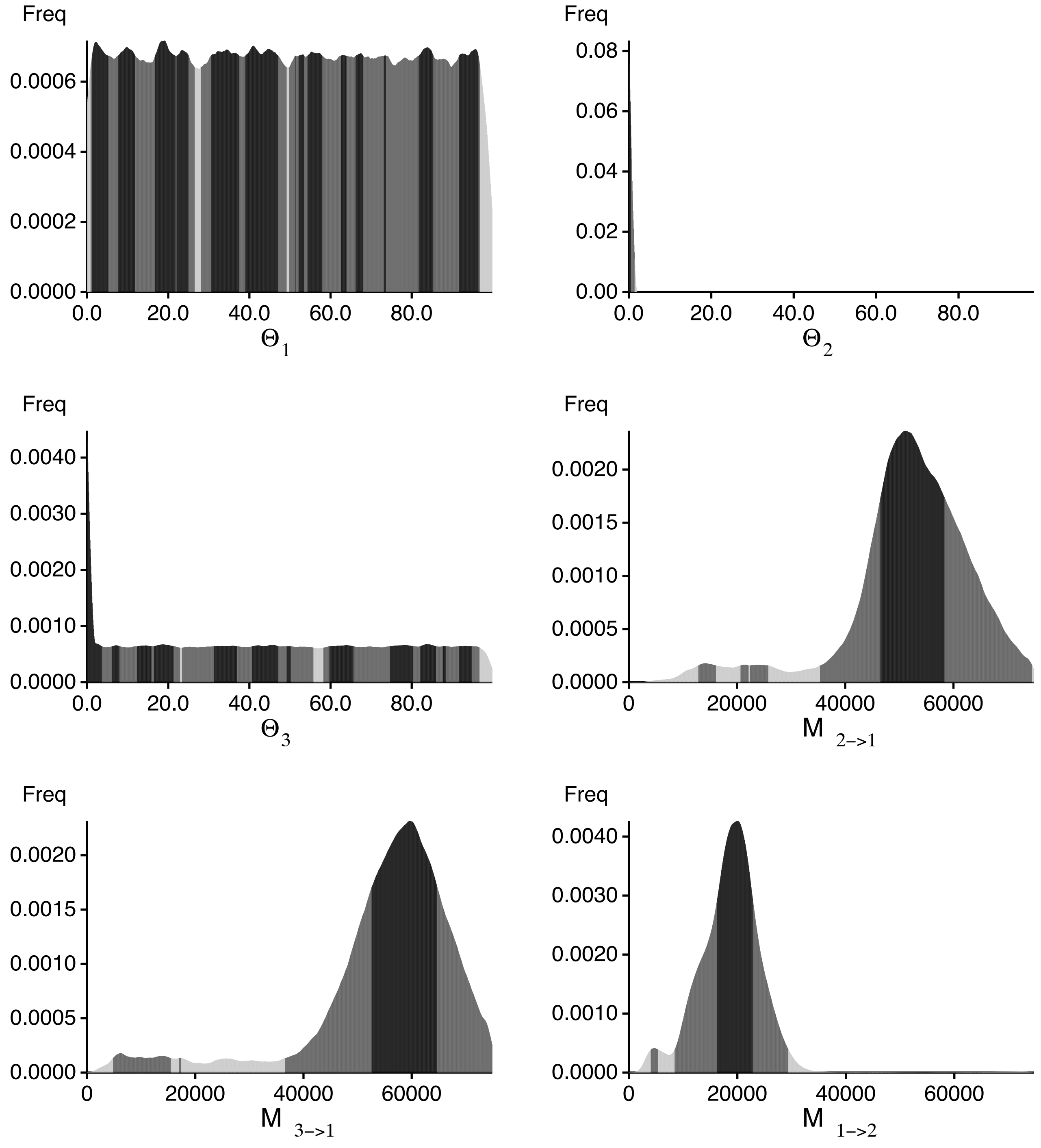

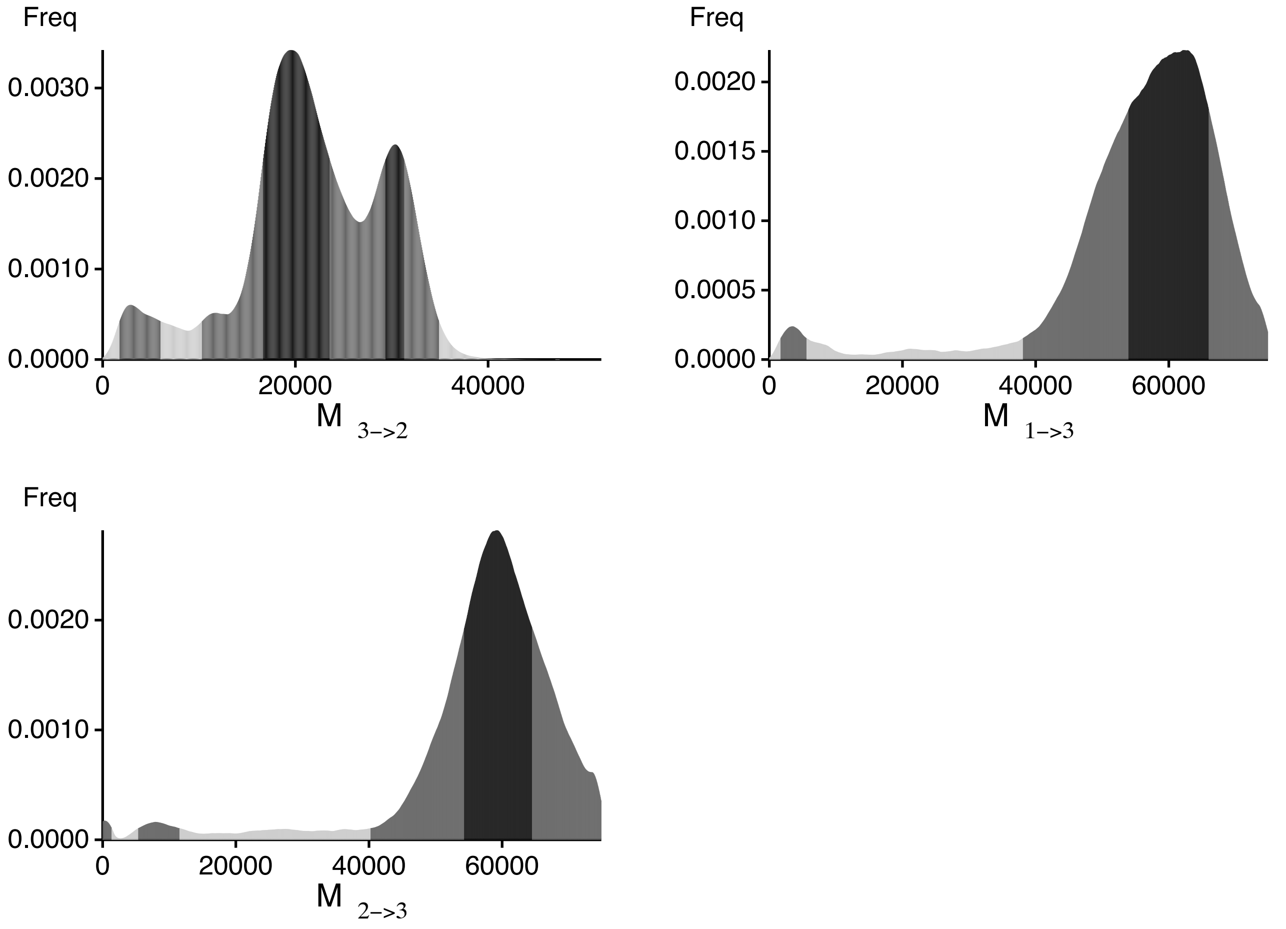
Freq
0.0030
0.0020
0.0010
0.0000
0
20000
40000
M 3->2
Freq
0.0020
0.0015
0.0010
0.0005
0.0000
0
20000
40000
60000
M 1->3
Freq
0.0020
0.0010
0.0000
0
20000
40000
60000
M 2->3

Log-Probability of the data given the model (marginal likelihood)

Use this value for Bayes factor calculations:
BF = Exp[ln(Prob(D I thisModel) - ln(Prob(D I otherModel)
or as LBF = 2 (ln(Prob(D I thisModel) - ln(Prob(D I otherModel))
shows the support for thisModel]

Method	ln(Prob(DIModel))	Notes
Thermodynamic integration	-2663.662265	(1a)
	-2662.929522	(1b)
Harmonic mean	-2518.607791	(2)

(1a, 1b and 2) is an approximation to the marginal likelihood, make sure the program run long enough!
(1a, 1b) and (2) should give a similar result, (2) is considered more
crude than (1), but (1) needs heating with several well-spaced chains,
(1b) is using a Bezier-curve to get better approximations for runs with low number
of heated chains

Acceptance ratios for all parameters and the genealogies

Parameter	Accepted changes	Ratio
Θ_1	417139/417139	1.00000
Θ_2	416479/416479	1.00000
Θ_3	416563/416563	1.00000
$M_{2->1}$	416045/416045	1.00000
$M_{3->1}$	417166/417166	1.00000
$M_{1->2}$	416343/416343	1.00000
$M_{3->2}$	417056/417056	1.00000
$M_{1->3}$	415658/415658	1.00000
$M_{2->3}$	415887/415887	1.00000
Genealogies	304248/2502671	0.12157

MCMC-Autocorrelation and Effective MCMC Sample Size

Parameter	Autocorrelation	Effective Sampe Size
Θ_1	0.01312	48705.34
Θ_2	0.68170	9463.51
Θ_3	0.03811	46329.24
$M_{2->1}$	0.84581	4176.90
$M_{3->1}$	0.88221	3129.10
$M_{1->2}$	0.84882	4088.65
$M_{3->2}$	0.93664	1635.93
$M_{1->3}$	0.88135	3153.38
$M_{2->3}$	0.87512	3329.86
Ln[Prob(D|G)]	0.53563	15119.74

Example: sequence data set wit two loci [simula

MIGRATION RATE AND POPULATION SIZE ESTIMATION
using the coalescent and maximum likelihood or Bayesian inference
Migrate-n version 3.2.6 [1776]
Program started at Tue Feb 5 16:39:18 2013
Program finished at Wed Feb 6 11:20:42 2013

Options

Datatype: DNA sequence data

Inheritance scalers in use for Thetas: 1.00
[Each Theta uses the (true) ineritance scalar of the first locus as a reference]
Random number seed: (with internal timer) 160670253
Start parameters:

Theta values were generated from the FST-calculation

M values were generated from the FST-calculation

Connection type matrix:
where m = average (average over a group of Thetas or M,
s = symmetric M, S = symmetric 4Nm, 0 = zero, and not estimated,
* = free to vary, Thetas are on diagonal

Population	1	2	3
1 BAJA	*	*	*
2 MIDR	*	*	*
3 MAIN	*	*	*

Order of parameters:

1	Θ_1	<displayed>
2	Θ_2	<displayed>
3	Θ_3	<displayed>
4	$M_{2->1}$	<displayed>
5	$M_{3->1}$	<displayed>
6	$M_{1->2}$	<displayed>

7	$M_{3->2}$	<displayed>
8	$M_{1->3}$	<displayed>
9	$M_{2->3}$	<displayed>

Mutation rate among loci: Mutation rate is constant

Analysis strategy: Bayesian inference

Proposal distributions for parameter

Parameter	Proposal
Theta	Slice sampling
M	Slice sampling

Prior distribution for parameter

Parameter	Prior	Minimum	Mean*	Maximum	Delta	Bins
Theta	Uniform	0.000000	50.000000	100.000000	10.000000	1500
M	Uniform	0.000000	37500.000000	75000.000000	7500.000000	1500

Markov chain settings:	Long chain
Number of chains	1
Recorded steps [a]	50000
Increment (record every x step [b]	100
Number of concurrent chains (replicates) [c]	1
Visited (sampled) parameter values [a*b*c]	5000000
Number of discard trees per chain (burn-in)	2500000

Multiple Markov chains:

Static heating scheme 20 chains with temperatures

5 2.40 2.10 2.00 1.95 1.80 1.65 1.50 1.40 1.25 1.10 1.00

Swapping interval is 1

Print options:	
Data file:	MRO_NGulf_direc.seq
Output file:	MRO_NGulf_subset_run3
Posterior distribution raw histogram file:	bayesfile
Print data:	No
Print genealogies [only some for some data type]:	None

Data summary

Datatype:	Sequence data
Number of loci:	1

Population	Locus	Gene copies
1 BAJA	1	30
2 MIDR	1	30
3 MAIN	1	30
Total of all populations	1	90

Bayesian Analysis: Posterior distribution table

Locus	Parameter	2.5%	25.0%	Mode	75.0%	97.5%	Median	Mean
1	Θ_1	51.93333	96.53333	98.23333	99.13333	99.26667	50.56667	50.18251
1	Θ_2	0.00000	0.00000	0.03333	2.73333	26.86667	45.10000	49.09784
1	Θ_3	0.00000	0.00000	0.03333	0.53333	1.40000	0.56667	0.22058
1	$M_{2->1}$	40250.0	53850.0	59175.0	63400.0	75000.0	57675.0	54331.2
1	$M_{3->1}$	42900.0	54900.0	59375.0	64350.0	75000.0	59125.0	55467.9
1	$M_{1->2}$	41550.0	54850.0	60425.0	64300.0	75000.0	58475.0	55218.8
1	$M_{3->2}$	39050.0	51650.0	55625.0	60550.0	74700.0	56075.0	54041.8
1	$M_{1->3}$	9250.0	17400.0	19775.0	22450.0	27250.0	19375.0	18059.5
1	$M_{2->3}$	12750.0	19950.0	22425.0	24750.0	35100.0	24025.0	23236.4

Bayesian Analysis: Posterior distribution over all loci

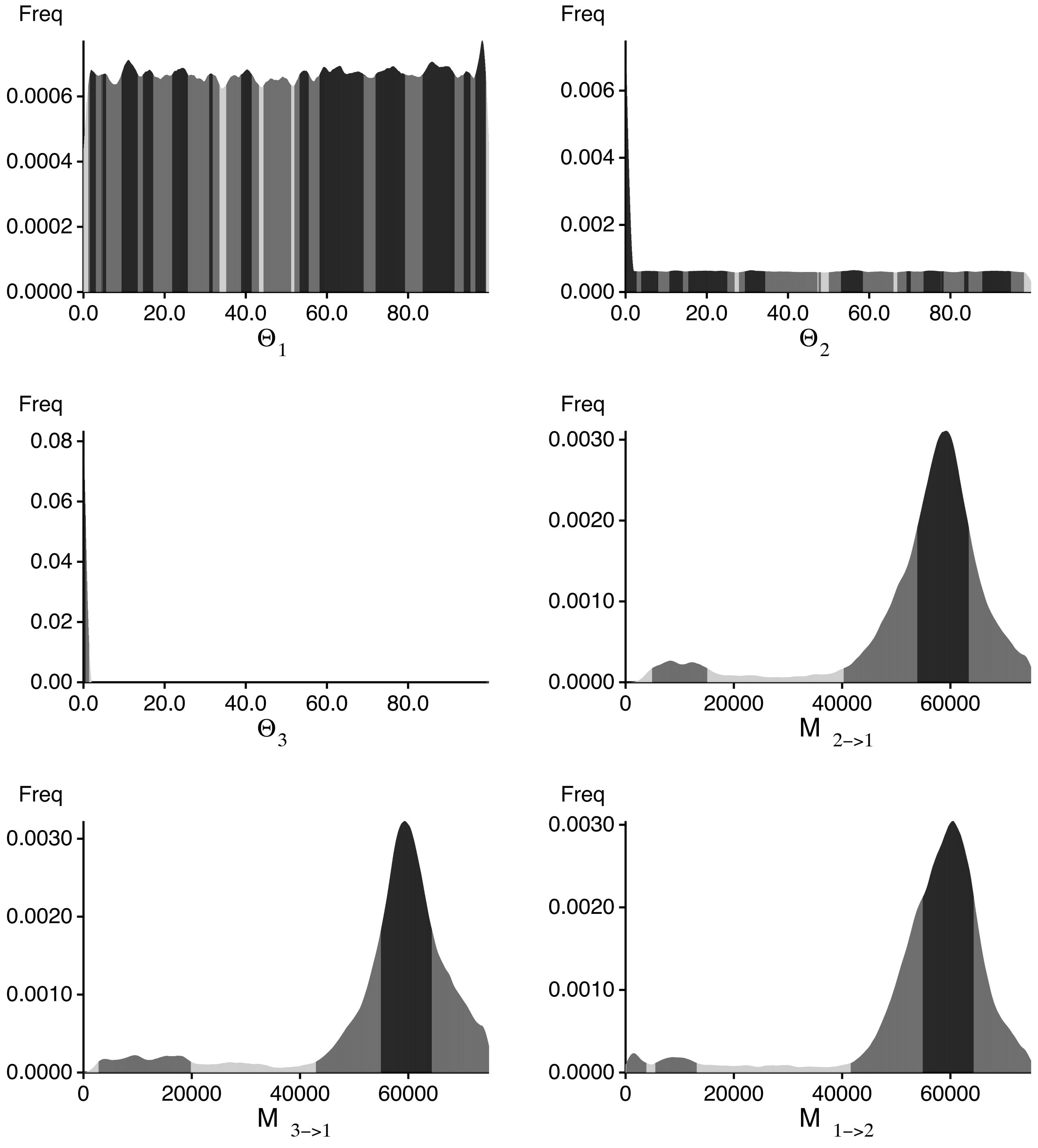

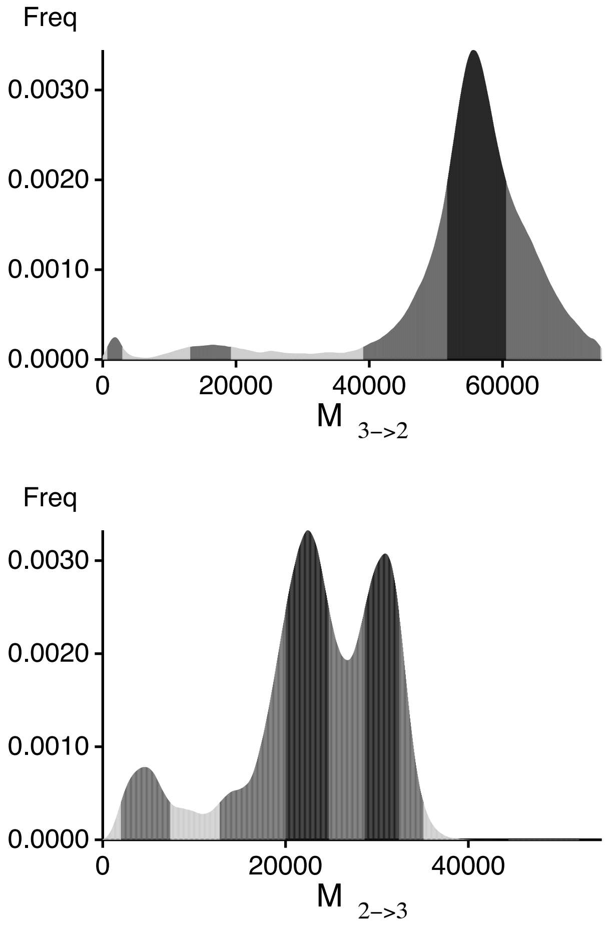
Freq
0.0030
0.0020
0.0010
0.0000
0
20000
40000
60000
M 3->2
Freq
0.006
0.004
0.002
0.000
0
10000
20000
30000
40000
M 1->3
Freq
0.0030
0.0020
0.0010
0.0000
0
20000
40000
M 2->3

Log-Probability of the data given the model (marginal likelihood)

Use this value for Bayes factor calculations:
BF = Exp[ln(Prob(D I thisModel) - ln(Prob(D I otherModel)
or as LBF = 2 (ln(Prob(D I thisModel) - ln(Prob(D I otherModel))
shows the support for thisModel]

Method	ln(Prob(DIModel))	Notes
Thermodynamic integration	-2646.847888	(1a)
	-2645.725295	(1b)
Harmonic mean	-2522.695382	(2)

(1a, 1b and 2) is an approximation to the marginal likelihood, make sure the program run long enough!
(1a, 1b) and (2) should give a similar result, (2) is considered more
crude than (1), but (1) needs heating with several well-spaced chains,
(1b) is using a Bezier-curve to get better approximations for runs with low number
of heated chains

Acceptance ratios for all parameters and the genealogies

Parameter	Accepted changes	Ratio
Θ_1	417549/417549	1.00000
Θ_2	416600/416600	1.00000
Θ_3	416315/416315	1.00000
$M_{2->1}$	415455/415455	1.00000
$M_{3->1}$	416012/416012	1.00000
$M_{1->2}$	415927/415927	1.00000
$M_{3->2}$	416774/416774	1.00000
$M_{1->3}$	416409/416409	1.00000
$M_{2->3}$	416620/416620	1.00000
Genealogies	252121/2502120	0.10076

MCMC-Autocorrelation and Effective MCMC Sample Size

Parameter	Autocorrelation	Effective Sampe Size
Θ_1	0.00913	49094.88
Θ_2	0.05775	44539.92
Θ_3	0.67196	9810.01
$M_{2->1}$	0.89595	2744.02
$M_{3->1}$	0.92465	1957.50
$M_{1->2}$	0.90275	2555.41
$M_{3->2}$	0.88179	3140.98
$M_{1->3}$	0.90244	2564.16
$M_{2->3}$	0.94632	1378.94
Ln[Prob(D|G)]	0.46867	18088.71

Warnings

You should most likely rerun your analysis after improving run parameters.

Param 1 (Locus 1): Upper prior boundary seems too low!
