# Supplementary material for: Asymmetric connectivity of spawning aggregations of a commercially important marine fish using a multidisciplinary approach
Source: PeerJ. 2014 Aug 7;2:e511. doi: 10.7717/peerj.511 (PMC4137664; doi:10.7717/peerj.511)
Supplement: File S2 — Detailed results from three independent runs with Migrate-n software for a model of gene flow from Baja across the Midriff Islands towards Sonora. [file peerj-02-511-s009.pdf]

Example: sequence data set wit two loci [simula

MIGRATION RATE AND POPULATION SIZE ESTIMATION
using the coalescent and maximum likelihood or Bayesian inference
Migrate-n version 3.2.16 [1920]
Compiled for a SYMMETRIC MULTIPROCESSORS
Program started at Tue Jan 29 09:43:29 2013
Program finished at Tue Jan 29 11:41:40 2013

Options

Datatype: DNA sequence data

Inheritance scalers in use for Thetas: 1.00
[Each Theta uses the (true) ineritance scalar of the first locus as a reference]
Random number seed: (with internal timer) 1352947789
Start parameters:

Theta values were generated from the FST-calculation

M values were generated from the FST-calculation

Connection type matrix:
where m = average (average over a group of Thetas or M,
s = symmetric M, S = symmetric 4Nm, 0 = zero, and not estimated,
* = free to vary, Thetas are on diagonal

Population	1	2	3
1 BAJA	*	0	0
2 MIDR	*	*	0
3 MAIN	*	*	*

Order of parameters:

1	Θ_1	<displayed>
2	Θ_2	<displayed>
3	Θ_3	<displayed>
6	$M_{1->2}$	<displayed>
8	$M_{1->3}$	<displayed>

9	$M_{2->3}$	<displayed>

Mutation rate among loci: Mutation rate is constant

Analysis strategy: Bayesian inference

Proposal distributions for parameter

Parameter	Proposal
Theta	Slice sampling
M	Slice sampling

Prior distribution for parameter

Parameter	Prior	Minimum	Mean*	Maximum	Delta	Bins
Theta	Uniform	0.000000	50.000000	100.000000	10.000000	1500
M	Uniform	0.000000	37500.000000	75000.000000	7500.000000	1500

Markov chain settings:	Long chain
Number of chains	1
Recorded steps [a]	50000
Increment (record every x step [b]	100
Number of concurrent chains (replicates) [c]	1
Visited (sampled) parameter values [a*b*c]	5000000
Number of discard trees per chain (burn-in)	2500000

Multiple Markov chains:

Static heating scheme 20 chains with temperatures

5 2.40 2.10 2.00 1.95 1.80 1.65 1.50 1.40 1.25 1.10 1.00

Swapping interval is 1

Print options:	
Data file:	MRO_NGulf_direc.seq
Output file:	MRO_NGulf_Direc2_run1
Posterior distribution raw histogram file:	bayesfile
Print data:	No
Print genealogies [only some for some data type]:	None

Data summary

Datatype:	Sequence data
Number of loci:	1

Population	Locus	Gene copies
1 BAJA	1	30
2 MIDR	1	30
3 MAIN	1	30
Total of all populations	1	90

Bayesian Analysis: Posterior distribution table

Locus	Parameter	2.5%	25.0%	Mode	75.0%	97.5%	Median	Mean
1	Θ_1	0.00000	0.00000	0.03333	0.53333	1.40000	0.56667	0.00736
1	Θ_2	1.00000	40.06667	45.83333	47.20000	48.00000	49.63334	49.98975
1	Θ_3	81.20000	97.06667	98.30000	98.93333	99.13333	50.03333	49.93806
1	$M_{1\text{->}2}$	4050.0	6800.0	8675.0	10850.0	17050.0	9525.0	10032.4
1	$M_{1\text{->}3}$	3200.0	10550.0	18775.0	33050.0	65450.0	28025.0	30690.1
1	$M_{2\text{->}3}$	15450.0	30650.0	48975.0	56750.0	74900.0	44375.0	44198.1

Bayesian Analysis: Posterior distribution over all loci

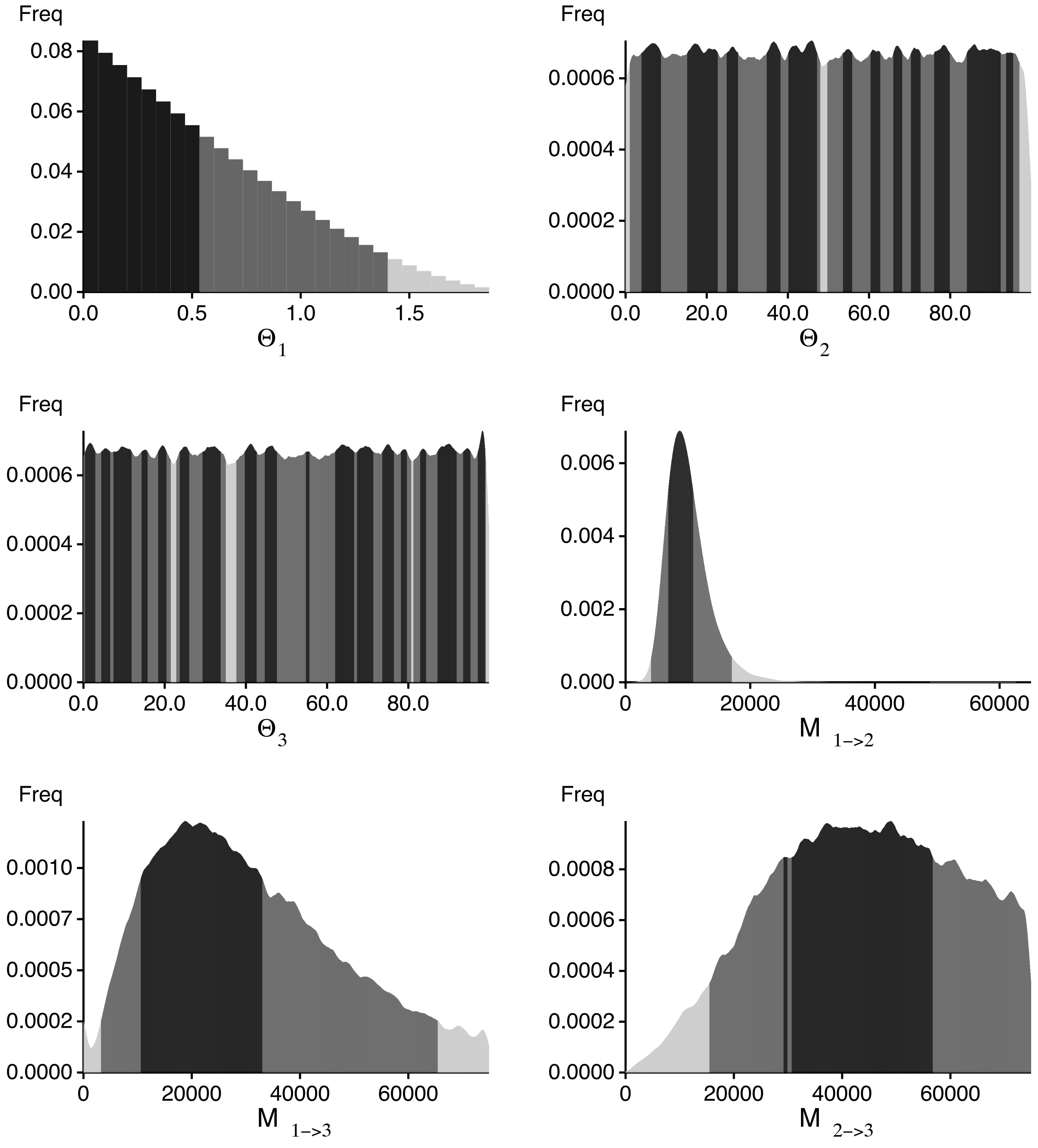

Log-Probability of the data given the model (marginal likelihood)

Use this value for Bayes factor calculations:
BF = Exp[ln(Prob(D I thisModel) - ln(Prob(D I otherModel)
or as LBF = 2 (ln(Prob(D I thisModel) - ln(Prob(D I otherModel))
shows the support for thisModel]

Method	ln(Prob(DIModel))	Notes
Thermodynamic integration	-2661.014048	(1a)
	-2658.636834	(1b)
Harmonic mean	-2513.071104	(2)

(1a, 1b and 2) is an approximation to the marginal likelihood, make sure the program run long enough!
(1a, 1b) and (2) should give a similar result, (2) is considered more
crude than (1), but (1) needs heating with several well-spaced chains,
(1b) is using a Bezier-curve to get better approximations for runs with low number
of heated chains

Acceptance ratios for all parameters and the genealogies

Parameter	Accepted changes	Ratio
Θ_1	624587/624587	1.00000
Θ_2	625395/625395	1.00000
Θ_3	625200/625200	1.00000
$M_{1->2}$	624409/624409	1.00000
$M_{1->3}$	625542/625542	1.00000
$M_{2->3}$	626232/626232	1.00000
Genealogies	493698/2498912	0.19757

MCMC-Autocorrelation and Effective MCMC Sample Size

Parameter	Autocorrelation	Effective Sampe Size
Θ_1	0.36828	23084.65
Θ_2	0.00592	49411.09
Θ_3	-0.00749	50755.05
$M_{1->2}$	0.50259	16551.69
$M_{1->3}$	0.45867	18555.64
$M_{2->3}$	0.48218	17468.08
Ln[Prob(D|G)]	0.53727	15050.49

Potential Problems

This section reports potential problems with your run, but such reporting is often not very accurate. Whith many parameters in a multilocus analysis, it is very common that some parameters for some loci will not be very informative, triggering suggestions (for example to increase the prior range) that are not sensible. This suggestion tool will improve with time, therefore do not blindly follow its suggestions. If some parameters are flagged, inspect the tables carefully and judge wether an action is required. For example, if you run a Bayesian inference with sequence data, for macroscopic species there is rarely the need to increase the prior for Theta beyond 0.1; but if you use microsatellites it is rather common that your prior distribution for Theta should have a range from 0.0 to 100 or more. With many populations (>3) it is also very common that some migration routes are estimated poorly because the data contains little or no information for that route. Increasing the range will not help in such situations, reducing number of parameters may help in such situations.

Param 3 (Locus 1): Upper prior boundary seems too low!

Example: sequence data set wit two loci [simula

MIGRATION RATE AND POPULATION SIZE ESTIMATION
using the coalescent and maximum likelihood or Bayesian inference
Migrate-n version 3.2.16 [1920]
Compiled for a SYMMETRIC MULTIPROCESSORS
Program started at Tue Jan 29 15:26:57 2013
Program finished at Thu Jan 31 09:59:52 2013

Options

Datatype:	DNA sequence data

Inheritance scalers in use for Thetas: 1.00
[Each Theta uses the (true) ineritance scalar of the first locus as a reference]

Random number seed:	(with internal timer)	996106619

Start parameters:

Theta values were generated	from the FST-calculation
M values were generated	from the FST-calculation

Connection type matrix:
where m = average (average over a group of Thetas or M,
s = symmetric M, S = symmetric 4Nm, 0 = zero, and not estimated,
* = free to vary, Thetas are on diagonal

Population	1	2	3
1 BAJA	*	0	0
2 MIDR	*	*	0
3 MAIN	*	*	*

Order of parameters:

1	Θ_1	<displayed>
2	Θ_2	<displayed>
3	Θ_3	<displayed>
6	$M_{1->2}$	<displayed>
8	$M_{1->3}$	<displayed>

9 $M_{2->3}$ <displayed>

Mutation rate among loci: Mutation rate is constant

Analysis strategy: Bayesian inference

Proposal distributions for parameter

Parameter	Proposal
Theta	Slice sampling
M	Slice sampling

Prior distribution for parameter

Parameter	Prior	Minimum	Mean*	Maximum	Delta	Bins
Theta	Uniform	0.000000	50.000000	100.000000	10.000000	1500
M	Uniform	0.000000	37500.000000	75000.000000	7500.000000	1500

Markov chain settings: Long chain

Number of chains 1

Recorded steps [a] 50000

Increment (record every x step [b] 100

Number of concurrent chains (replicates) [c] 1

Visited (sampled) parameter values [a*b*c] 5000000

Number of discard trees per chain (burn-in) 2500000

Multiple Markov chains:

Static heating scheme 20 chains with temperatures

5 2.40 2.10 2.00 1.95 1.80 1.65 1.50 1.40 1.25 1.10 1.00

Swapping interval is 1

Print options:

Data file: MRO_NGulf_direc.seq

Output file: MRO_NGulf_Direc2_run2

Posterior distribution raw histogram file: bayesfile

Print data: No

Print genealogies [only some for some data type]: None

Data summary

Datatype:	Sequence data
Number of loci:	1

Population	Locus	Gene copies
1 BAJA	1	30
2 MIDR	1	30
3 MAIN	1	30
Total of all populations	1	90

Bayesian Analysis: Posterior distribution table

Locus	Parameter	2.5%	25.0%	Mode	75.0%	97.5%	Median	Mean
1	Θ_1	0.00000	0.00000	0.03333	0.53333	1.40000	0.56667	0.00752
1	Θ_2	3.00000	25.60000	26.96667	29.40000	46.06667	50.10000	50.09869
1	Θ_3	29.26667	30.13333	32.03333	33.80000	44.66667	49.90000	50.03272
1	$M_{1\text{->}2}$	4100.0	6700.0	8525.0	10700.0	16700.0	9425.0	9930.3
1	$M_{1\text{->}3}$	2550.0	10150.0	20275.0	30900.0	61850.0	26125.0	28985.9
1	$M_{2\text{->}3}$	15750.0	34950.0	46125.0	60650.0	75000.0	46075.0	45358.6

Bayesian Analysis: Posterior distribution over all loci

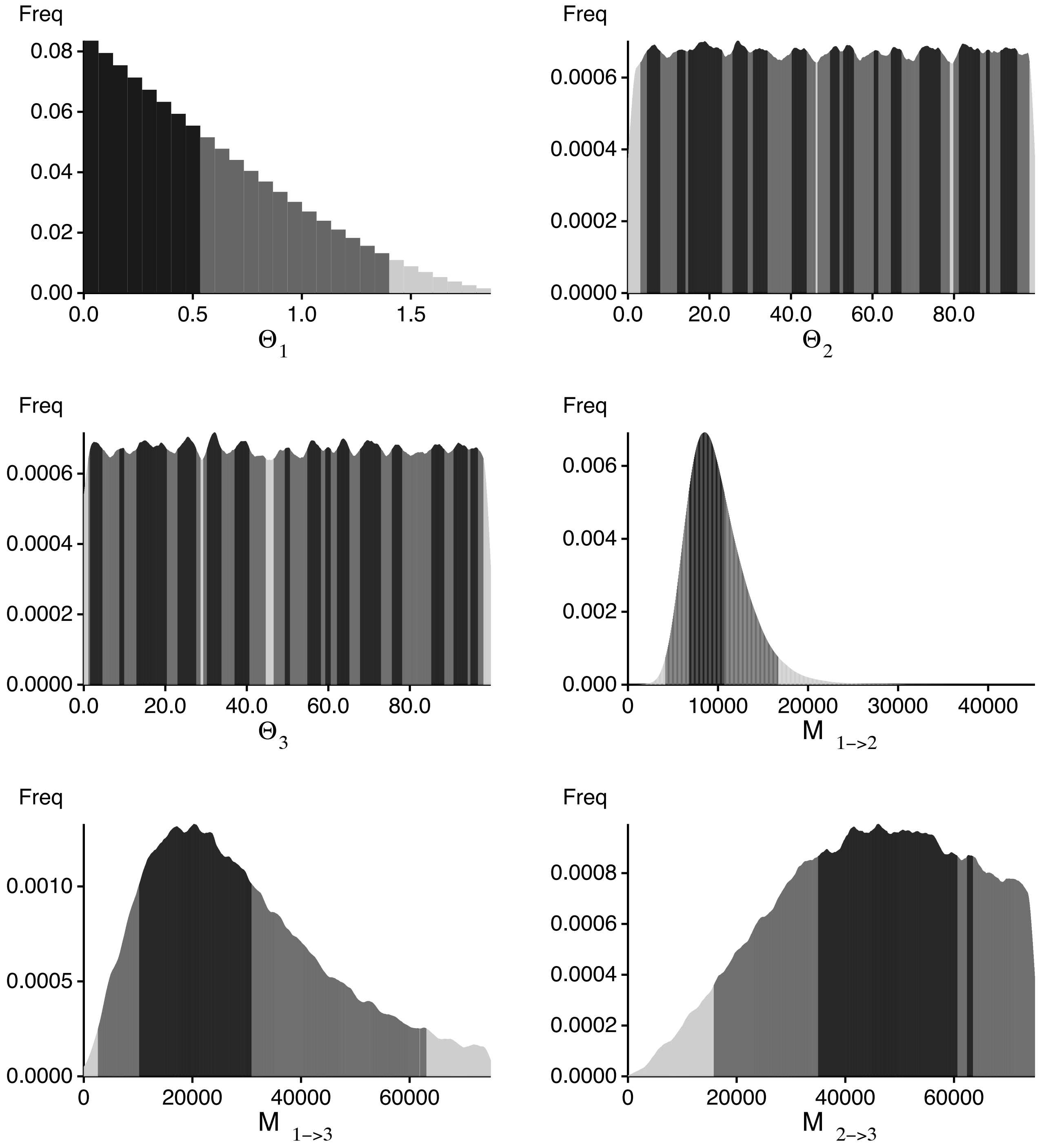

Log-Probability of the data given the model (marginal likelihood)

Use this value for Bayes factor calculations:
BF = Exp[ln(Prob(D I thisModel) - ln(Prob(D I otherModel)
or as LBF = 2 (ln(Prob(D I thisModel) - ln(Prob(D I otherModel))
shows the support for thisModel]

Method	ln(Prob(DIModel))	Notes
Thermodynamic integration	-2660.566050	(1a)
	-2658.266866	(1b)
Harmonic mean	-2517.504887	(2)

(1a, 1b and 2) is an approximation to the marginal likelihood, make sure the program run long enough!
(1a, 1b) and (2) should give a similar result, (2) is considered more
crude than (1), but (1) needs heating with several well-spaced chains,
(1b) is using a Bezier-curve to get better approximations for runs with low number
of heated chains

Acceptance ratios for all parameters and the genealogies

Parameter	Accepted changes	Ratio
Θ_1	624973/624973	1.00000
Θ_2	626521/626521	1.00000
Θ_3	625271/625271	1.00000
$M_{1->2}$	623554/623554	1.00000
$M_{1->3}$	625061/625061	1.00000
$M_{2->3}$	624369/624369	1.00000
Genealogies	491264/2501306	0.19640

MCMC-Autocorrelation and Effective MCMC Sample Size

Parameter	Autocorrelation	Effective Sampe Size
Θ_1	0.40862	20991.32
Θ_2	0.00082	49917.72
Θ_3	0.00272	49728.94
$M_{1->2}$	0.47700	17704.76
$M_{1->3}$	0.47190	17939.59
$M_{2->3}$	0.50422	16479.62
Ln[Prob(D|G)]	0.54818	14591.84

Potential Problems

This section reports potential problems with your run, but such reporting is often not very accurate. Whith many parameters in a multilocus analysis, it is very common that some parameters for some loci will not be very informative, triggering suggestions (for example to increase the prior range) that are not sensible. This suggestion tool will improve with time, therefore do not blindly follow its suggestions. If some parameters are flagged, inspect the tables carefully and judge wether an action is required. For example, if you run a Bayesian inference with sequence data, for macroscopic species there is rarely the need to increase the prior for Theta beyond 0.1; but if you use microsatellites it is rather common that your prior distribution for Theta should have a range from 0.0 to 100 or more. With many populations (>3) it is also very common that some migration routes are estimated poorly because the data contains little or no information for that route. Increasing the range will not help in such situations, reducing number of parameters may help in such situations.

No warning was recorded during the run

Example: sequence data set wit two loci [simula

MIGRATION RATE AND POPULATION SIZE ESTIMATION
using the coalescent and maximum likelihood or Bayesian inference
Migrate-n version 3.2.16 [1920]
Compiled for a SYMMETRIC MULTIPROCESSORS
Program started at Thu Jan 31 23:00:16 2013
Program finished at Fri Feb 1 00:58:35 2013

Options

Datatype:	DNA sequence data

Inheritance scalers in use for Thetas: 1.00
[Each Theta uses the (true) ineritance scalar of the first locus as a reference]

Random number seed:	(with internal timer)	599348375

Start parameters:

Theta values were generated	from the FST-calculation
M values were generated	from the FST-calculation

Connection type matrix:
where m = average (average over a group of Thetas or M,
s = symmetric M, S = symmetric 4Nm, 0 = zero, and not estimated,
* = free to vary, Thetas are on diagonal

Population	1	2	3
1 BAJA	*	0	0
2 MIDR	*	*	0
3 MAIN	*	*	*

Order of parameters:

1	Θ_1	<displayed>
2	Θ_2	<displayed>
3	Θ_3	<displayed>
6	$M_{1->2}$	<displayed>
8	$M_{1->3}$	<displayed>

9	$M_{2->3}$	<displayed>

Mutation rate among loci: Mutation rate is constant

Analysis strategy: Bayesian inference

Proposal distributions for parameter

Parameter	Proposal
Theta	Slice sampling
M	Slice sampling

Prior distribution for parameter

Parameter	Prior	Minimum	Mean*	Maximum	Delta	Bins
Theta	Uniform	0.000000	50.000000	100.000000	10.000000	1500
M	Uniform	0.000000	37500.000000	75000.000000	7500.000000	1500

Markov chain settings:	Long chain
Number of chains	1
Recorded steps [a]	50000
Increment (record every x step [b]	100
Number of concurrent chains (replicates) [c]	1
Visited (sampled) parameter values [a*b*c]	5000000
Number of discard trees per chain (burn-in)	2500000

Multiple Markov chains:
Static heating scheme 20 chains with temperatures
5 2.40 2.10 2.00 1.95 1.80 1.65 1.50 1.40 1.25 1.10 1.00
Swapping interval is 1

Print options:	
Data file:	MRO_NGulf_direc.seq
Output file:	MRO_NGulf_Direc2_run1
Posterior distribution raw histogram file:	bayesfile
Print data:	No
Print genealogies [only some for some data type]:	None

Data summary

Datatype:	Sequence data
Number of loci:	1

Population	Locus	Gene copies
1 BAJA	1	30
2 MIDR	1	30
3 MAIN	1	30
Total of all populations	1	90

Bayesian Analysis: Posterior distribution table

Locus	Parameter	2.5%	25.0%	Mode	75.0%	97.5%	Median	Mean
1	Θ_1	0.00000	0.00000	0.03333	0.53333	1.40000	0.56667	0.00748
1	Θ_2	0.00000	0.00000	0.03333	4.73333	29.73333	49.70000	49.80238
1	Θ_3	1.00000	6.60000	8.90000	11.26667	43.80000	49.76667	49.90507
1	$M_{1->2}$	4250.0	7000.0	8775.0	11000.0	17400.0	9725.0	10251.9
1	$M_{1->3}$	2950.0	10200.0	20975.0	32200.0	64450.0	27475.0	30287.4
1	$M_{2->3}$	14950.0	32600.0	45475.0	57850.0	75000.0	44625.0	44230.9

Bayesian Analysis: Posterior distribution over all loci

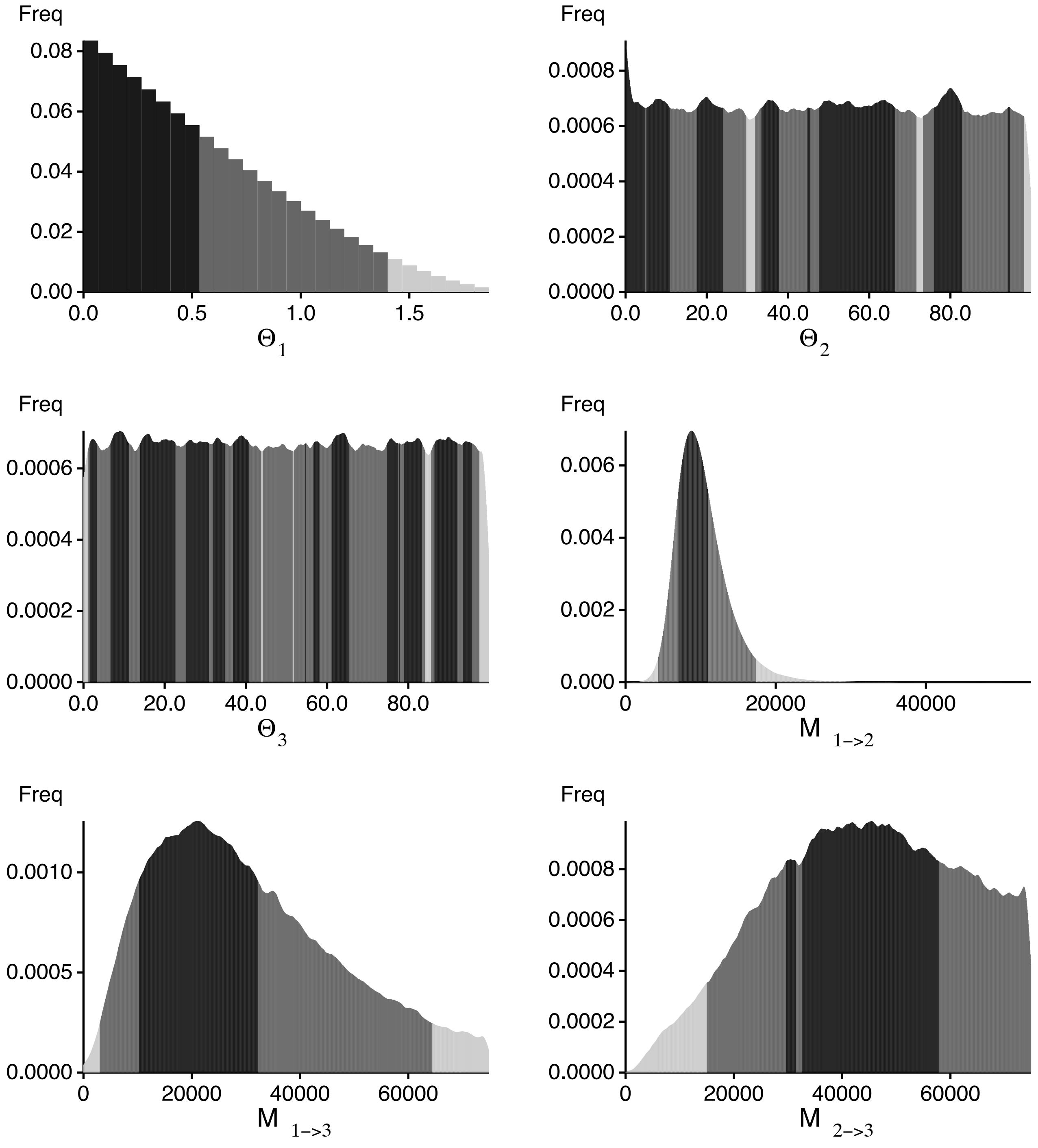

Log-Probability of the data given the model (marginal likelihood)

Use this value for Bayes factor calculations:
BF = Exp[ln(Prob(D I thisModel) - ln(Prob(D I otherModel)
or as LBF = 2 (ln(Prob(D I thisModel) - ln(Prob(D I otherModel))
shows the support for thisModel]

Method	ln(Prob(DIModel))	Notes
Thermodynamic integration	-2660.905427	(1a)
	-2658.464366	(1b)
Harmonic mean	-2515.794262	(2)

(1a, 1b and 2) is an approximation to the marginal likelihood, make sure the program run long enough!
(1a, 1b) and (2) should give a similar result, (2) is considered more
crude than (1), but (1) needs heating with several well-spaced chains,
(1b) is using a Bezier-curve to get better approximations for runs with low number
of heated chains

Acceptance ratios for all parameters and the genealogies

Parameter	Accepted changes	Ratio
Θ_1	625435/625435	1.00000
Θ_2	624793/624793	1.00000
Θ_3	625019/625019	1.00000
$M_{1->2}$	625290/625290	1.00000
$M_{1->3}$	624017/624017	1.00000
$M_{2->3}$	626584/626584	1.00000
Genealogies	492370/2499774	0.19697

MCMC-Autocorrelation and Effective MCMC Sample Size

Parameter	Autocorrelation	Effective Sampe Size
Θ_1	0.38129	22395.87
Θ_2	0.02018	48021.49
Θ_3	-0.00076	50076.32
$M_{1->2}$	0.47159	17953.89
$M_{1->3}$	0.47816	17651.48
$M_{2->3}$	0.50152	16599.39
Ln[Prob(D|G)]	0.53923	14967.64

Potential Problems

This section reports potential problems with your run, but such reporting is often not very accurate. Whith many parameters in a multilocus analysis, it is very common that some parameters for some loci will not be very informative, triggering suggestions (for example to increase the prior range) that are not sensible. This suggestion tool will improve with time, therefore do not blindly follow its suggestions. If some parameters are flagged, inspect the tables carefully and judge wether an action is required. For example, if you run a Bayesian inference with sequence data, for macroscopic species there is rarely the need to increase the prior for Theta beyond 0.1; but if you use microsatellites it is rather common that your prior distribution for Theta should have a range from 0.0 to 100 or more. With many populations (>3) it is also very common that some migration routes are estimated poorly because the data contains little or no information for that route. Increasing the range will not help in such situations, reducing number of parameters may help in such situations.

No warning was recorded during the run
