# Supplementary material for: Asymmetric connectivity of spawning aggregations of a commercially important marine fish using a multidisciplinary approach
Source: PeerJ. 2014 Aug 7;2:e511. doi: 10.7717/peerj.511 (PMC4137664; doi:10.7717/peerj.511)
Supplement: File S3 — Detailed results from three independent runs with Migrate-n software for a model of gene flow from Sonora across the Midriff Islands towards Baja. [file peerj-02-511-s010.pdf]

Example: sequence data set wit two loci [simula

MIGRATION RATE AND POPULATION SIZE ESTIMATION
using the coalescent and maximum likelihood or Bayesian inference
Migrate-n version 3.2.6 [1776]
Program started at Sun Jan 27 15:05:54 2013
Program finished at Sun Jan 27 16:28:46 2013

Options

Datatype: DNA sequence data

Inheritance scalers in use for Thetas: 1.00
[Each Theta uses the (true) ineritance scalar of the first locus as a reference]
Random number seed: (with internal timer) 1213855653
Start parameters:

Theta values were generated from the FST-calculation

M values were generated from the FST-calculation

Connection type matrix:
where m = average (average over a group of Thetas or M,
s = symmetric M, S = symmetric 4Nm, 0 = zero, and not estimated,
* = free to vary, Thetas are on diagonal

Population	1	2	3
1 BAJA	*	*	*
2 MIDR	0	*	*
3 MAIN	0	0	*

Order of parameters:

1	Θ_1	<displayed>
2	Θ_2	<displayed>
3	Θ_3	<displayed>
4	$M_{2->1}$	<displayed>
5	$M_{3->1}$	<displayed>
7	$M_{3->2}$	<displayed>

Mutation rate among loci: Mutation rate is constant

Analysis strategy: Bayesian inference

Proposal distributions for parameter

Parameter	Proposal
Theta	Slice sampling
M	Slice sampling

Prior distribution for parameter

Parameter	Prior	Minimum	Mean*	Maximum	Delta	Bins
Theta	Uniform	0.000000	50.000000	100.000000	10.000000	1500
M	Uniform	0.000000	37500.000000	75000.000000	7500.000000	1500

Markov chain settings: Long chain

Number of chains	1
Recorded steps [a]	50000
Increment (record every x step [b]	100
Number of concurrent chains (replicates) [c]	1
Visited (sampled) parameter values [a*b*c]	5000000
Number of discard trees per chain (burn-in)	2500000

Multiple Markov chains:

Static heating scheme 20 chains with temperatures

5 2.40 2.10 2.00 1.95 1.80 1.65 1.50 1.40 1.25 1.10 1.00

Swapping interval is 1

Print options:

Data file:	MRO_NGulf_direc.seq
Output file:	MRO_NGulf_Direc1_run1
Posterior distribution raw histogram file:	bayesfile
Print data:	No
Print genealogies [only some for some data type]:	None

Data summary

Datatype:	Sequence data
Number of loci:	1

Population	Locus	Gene copies
1 BAJA	1	30
2 MIDR	1	30
3 MAIN	1	30
Total of all populations	1	90

Bayesian Analysis: Posterior distribution table

Locus	Parameter	2.5%	25.0%	Mode	75.0%	97.5%	Median	Mean
1	Θ_1	18.86667	69.20000	71.56667	73.20000	92.66666	50.03333	50.09443
1	Θ_2	31.00000	46.53333	48.83333	50.46667	86.33334	49.83333	50.04533
1	Θ_3	0.00000	0.00000	0.03333	0.53333	1.40000	0.56667	0.00760
1	$M_{2->1}$	17550.0	35750.0	48825.0	60300.0	75000.0	46575.0	45951.8
1	$M_{3->1}$	2250.0	10350.0	18075.0	31050.0	61600.0	26125.0	28825.8
1	$M_{3->2}$	4300.0	7000.0	9025.0	11300.0	18100.0	10025.0	10559.0

Bayesian Analysis: Posterior distribution over all loci

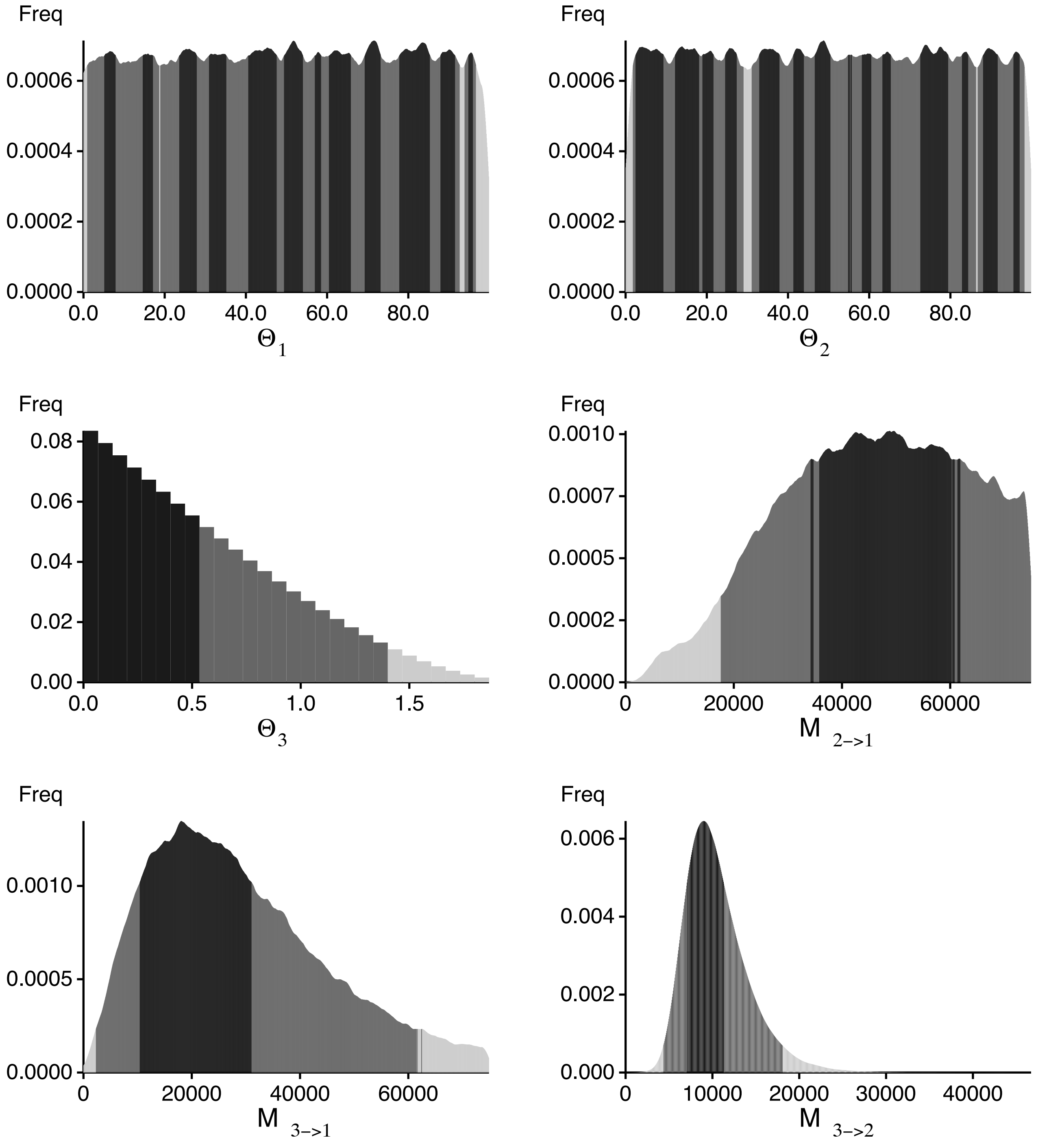

Log-Probability of the data given the model (marginal likelihood)

Use this value for Bayes factor calculations:
BF = Exp[ln(Prob(D I thisModel) - ln(Prob(D I otherModel)
or as LBF = 2 (ln(Prob(D I thisModel) - ln(Prob(D I otherModel))
shows the support for thisModel]

Method	ln(Prob(DIModel))	Notes
Thermodynamic integration	-3095.438191	(1a)
	-2974.327042	(1b)
Harmonic mean	-2529.205774	(2)

(1a, 1b and 2) is an approximation to the marginal likelihood, make sure the program run long enough!
(1a, 1b) and (2) should give a similar result, (2) is considered more
crude than (1), but (1) needs heating with several well-spaced chains,
(1b) is using a Bezier-curve to get better approximations for runs with low number
of heated chains

Acceptance ratios for all parameters and the genealogies

Parameter	Accepted changes	Ratio
Θ_1	625510/625510	1.00000
Θ_2	625293/625293	1.00000
Θ_3	623809/623809	1.00000
$M_{2->1}$	625566/625566	1.00000
$M_{3->1}$	623679/623679	1.00000
$M_{3->2}$	626240/626240	1.00000
Genealogies	488947/2500048	0.19558

MCMC-Autocorrelation and Effective MCMC Sample Size

Parameter	Autocorrelation	Effective Sampe Size
Θ_1	-0.00348	50349.57
Θ_2	0.00546	49457.23
Θ_3	0.37793	22572.61
$M_{2->1}$	0.49144	17049.07
$M_{3->1}$	0.45454	18750.11
$M_{3->2}$	0.44707	19105.27
Ln[Prob(D|G)]	0.52614	15524.72

Example: sequence data set wit two loci [simula

MIGRATION RATE AND POPULATION SIZE ESTIMATION
using the coalescent and maximum likelihood or Bayesian inference
Migrate-n version 3.2.6 [1776]
Program started at Tue Jan 29 09:30:51 2013
Program finished at Tue Jan 29 10:54:17 2013

Options

Datatype:	DNA sequence data

Inheritance scalers in use for Thetas: 1.00
[Each Theta uses the (true) ineritance scalar of the first locus as a reference]

Random number seed:	(with internal timer)	597500213

Start parameters:

Theta values were generated	from the FST-calculation
M values were generated	from the FST-calculation

Connection type matrix:
where m = average (average over a group of Thetas or M,
s = symmetric M, S = symmetric 4Nm, 0 = zero, and not estimated,
* = free to vary, Thetas are on diagonal

Population	1	2	3
1 BAJA	*	*	*
2 MIDR	0	*	*
3 MAIN	0	0	*

Order of parameters:

1	Θ_1	<displayed>
2	Θ_2	<displayed>
3	Θ_3	<displayed>
4	$M_{2->1}$	<displayed>
5	$M_{3->1}$	<displayed>
7	$M_{3->2}$	<displayed>

Mutation rate among loci: Mutation rate is constant

Analysis strategy: Bayesian inference

Proposal distributions for parameter

Parameter	Proposal
Theta	Slice sampling
M	Slice sampling

Prior distribution for parameter

Parameter	Prior	Minimum	Mean*	Maximum	Delta	Bins
Theta	Uniform	0.000000	50.000000	100.000000	10.000000	1500
M	Uniform	0.000000	37500.000000	75000.000000	7500.000000	1500

Markov chain settings: Long chain

Number of chains 1

Recorded steps [a] 50000

Increment (record every x step [b] 100

Number of concurrent chains (replicates) [c] 1

Visited (sampled) parameter values [a*b*c] 5000000

Number of discard trees per chain (burn-in) 2500000

Multiple Markov chains:

Static heating scheme 20 chains with temperatures

5 2.40 2.10 2.00 1.95 1.80 1.65 1.50 1.40 1.25 1.10 1.00

Swapping interval is 1

Print options:

Data file: MRO_NGulf_direc.seq

Output file: MRO_NGulf_Direc1_run2

Posterior distribution raw histogram file: bayesfile

Print data: No

Print genealogies [only some for some data type]: None

Data summary

Datatype:	Sequence data
Number of loci:	1

Population	Locus	Gene copies
1 BAJA	1	30
2 MIDR	1	30
3 MAIN	1	30
Total of all populations	1	90

Bayesian Analysis: Posterior distribution table

Locus	Parameter	2.5%	25.0%	Mode	75.0%	97.5%	Median	Mean
1	Θ_1	0.80000	9.53333	11.56667	16.60000	39.73333	50.10000	49.93845
1	Θ_2	5.00000	6.60000	8.83333	11.40000	22.86667	49.56667	49.90416
1	Θ_3	0.00000	0.00000	0.03333	0.53333	1.40000	0.56667	0.00759
1	$M_{2->1}$	19550.0	37400.0	43275.0	58350.0	74750.0	47275.0	46981.7
1	$M_{3->1}$	3200.0	11750.0	23925.0	35300.0	66150.0	29025.0	31721.9
1	$M_{3->2}$	4350.0	7100.0	9125.0	11400.0	18450.0	10075.0	10685.0

Bayesian Analysis: Posterior distribution over all loci

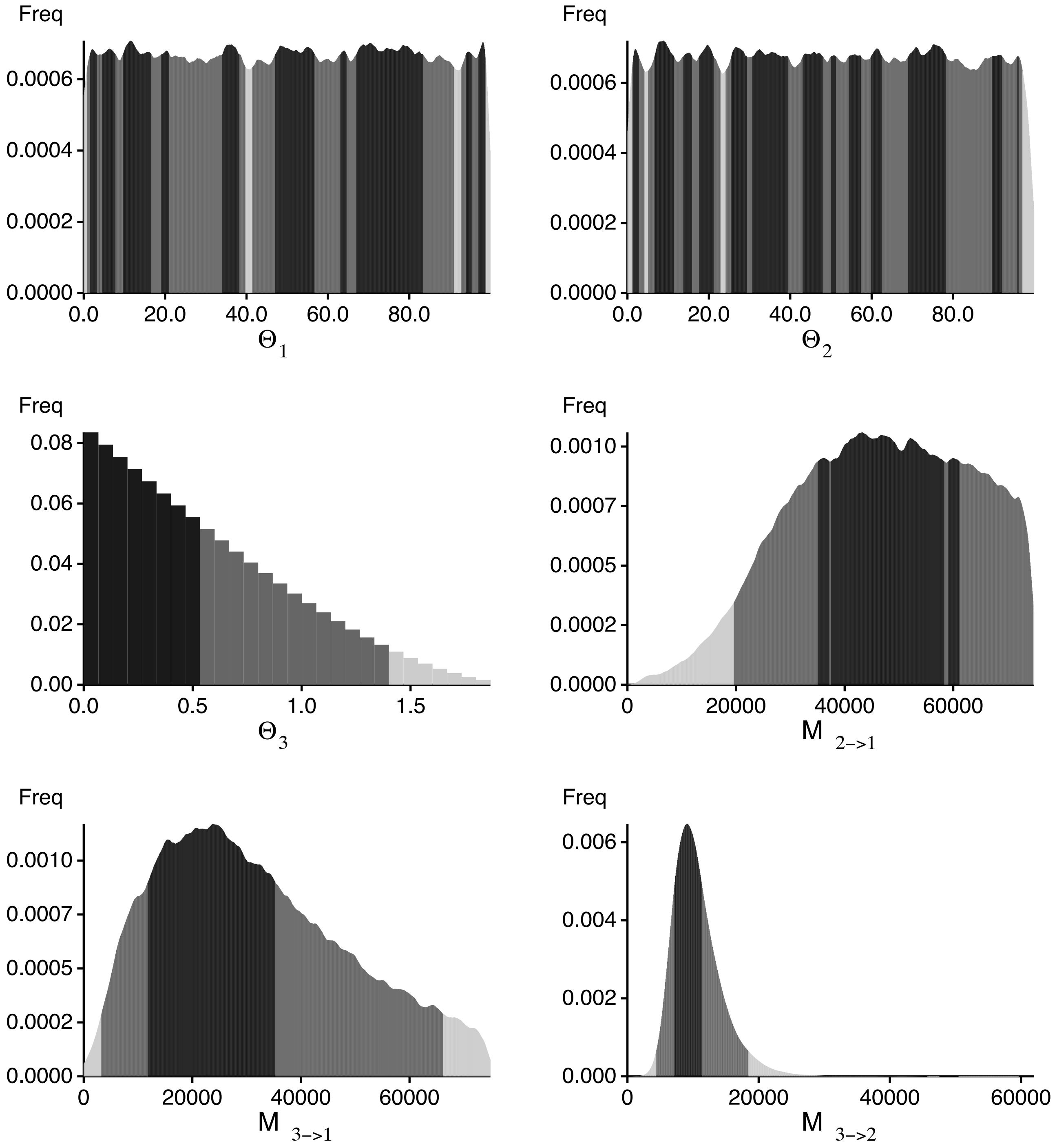

Log-Probability of the data given the model (marginal likelihood)

Use this value for Bayes factor calculations:
BF = Exp[ln(Prob(D I thisModel) - ln(Prob(D I otherModel)
or as LBF = 2 (ln(Prob(D I thisModel) - ln(Prob(D I otherModel))
shows the support for thisModel]

Method	ln(Prob(DIModel))	Notes
Thermodynamic integration	-3097.747293	(1a)
	-2976.408268	(1b)
Harmonic mean	-2550.572856	(2)

(1a, 1b and 2) is an approximation to the marginal likelihood, make sure the program run long enough!
(1a, 1b) and (2) should give a similar result, (2) is considered more
crude than (1), but (1) needs heating with several well-spaced chains,
(1b) is using a Bezier-curve to get better approximations for runs with low number
of heated chains

Acceptance ratios for all parameters and the genealogies

Parameter	Accepted changes	Ratio
Θ_1	625209/625209	1.00000
Θ_2	625116/625116	1.00000
Θ_3	625303/625303	1.00000
$M_{2->1}$	624999/624999	1.00000
$M_{3->1}$	624094/624094	1.00000
$M_{3->2}$	625270/625270	1.00000
Genealogies	488739/2501165	0.19540

MCMC-Autocorrelation and Effective MCMC Sample Size

Parameter	Autocorrelation	Effective Sampe Size
Θ_1	-0.00389	50390.38
Θ_2	0.01303	48713.73
Θ_3	0.36067	23493.21
$M_{2->1}$	0.42072	20386.82
$M_{3->1}$	0.47805	17656.85
$M_{3->2}$	0.47626	17738.80
Ln[Prob(D|G)]	0.55010	14512.05

Example: sequence data set wit two loci [simula

MIGRATION RATE AND POPULATION SIZE ESTIMATION
using the coalescent and maximum likelihood or Bayesian inference
Migrate-n version 3.2.6 [1776]
Program started at Tue Jan 29 15:29:48 2013
Program finished at Tue Jan 29 16:51:48 2013

Options

Datatype: DNA sequence data

Inheritance scalers in use for Thetas: 1.00
[Each Theta uses the (true) ineritance scalar of the first locus as a reference]
Random number seed: (with internal timer) 266474721
Start parameters:

Theta values were generated from the FST-calculation

M values were generated from the FST-calculation

Connection type matrix:
where m = average (average over a group of Thetas or M,
s = symmetric M, S = symmetric 4Nm, 0 = zero, and not estimated,
* = free to vary, Thetas are on diagonal

Population	1	2	3
1 BAJA	*	*	*
2 MIDR	0	*	*
3 MAIN	0	0	*

Order of parameters:

1	Θ_1	<displayed>
2	Θ_2	<displayed>
3	Θ_3	<displayed>
4	$M_{2->1}$	<displayed>
5	$M_{3->1}$	<displayed>
7	$M_{3->2}$	<displayed>

Mutation rate among loci: Mutation rate is constant

Analysis strategy: Bayesian inference

Proposal distributions for parameter

Parameter	Proposal
Theta	Slice sampling
M	Slice sampling

Prior distribution for parameter

Parameter	Prior	Minimum	Mean*	Maximum	Delta	Bins
Theta	Uniform	0.000000	50.000000	100.000000	10.000000	1500
M	Uniform	0.000000	37500.000000	75000.000000	7500.000000	1500

Markov chain settings: Long chain

Number of chains 1

Recorded steps [a] 50000

Increment (record every x step [b] 100

Number of concurrent chains (replicates) [c] 1

Visited (sampled) parameter values [a*b*c] 5000000

Number of discard trees per chain (burn-in) 2500000

Multiple Markov chains:

Static heating scheme 20 chains with temperatures

5 2.40 2.10 2.00 1.95 1.80 1.65 1.50 1.40 1.25 1.10 1.00

Swapping interval is 1

Print options:

Data file: MRO_NGulf_direc.seq

Output file: MRO_NGulf_Direc1_run3

Posterior distribution raw histogram file: bayesfile

Print data: No

Print genealogies [only some for some data type]: None

Data summary

Datatype:	Sequence data
Number of loci:	1

Population	Locus	Gene copies
1 BAJA	1	30
2 MIDR	1	30
3 MAIN	1	30
Total of all populations	1	90

Bayesian Analysis: Posterior distribution table

Locus	Parameter	2.5%	25.0%	Mode	75.0%	97.5%	Median	Mean
1	Θ_1	24.20000	73.86667	76.83334	85.13333	92.33334	50.10000	50.18420
1	Θ_2	62.26667	77.06667	79.03333	81.26667	93.33334	49.83333	50.01832
1	Θ_3	0.00000	0.00000	0.03333	0.53333	1.40000	0.56667	0.00757
1	$M_{2->1}$	15100.0	31150.0	42225.0	57600.0	75000.0	45325.0	44848.9
1	$M_{3->1}$	1350.0	9850.0	16825.0	31650.0	61350.0	25925.0	28507.9
1	$M_{3->2}$	4550.0	7350.0	9225.0	11500.0	17700.0	10175.0	10638.9

Bayesian Analysis: Posterior distribution over all loci

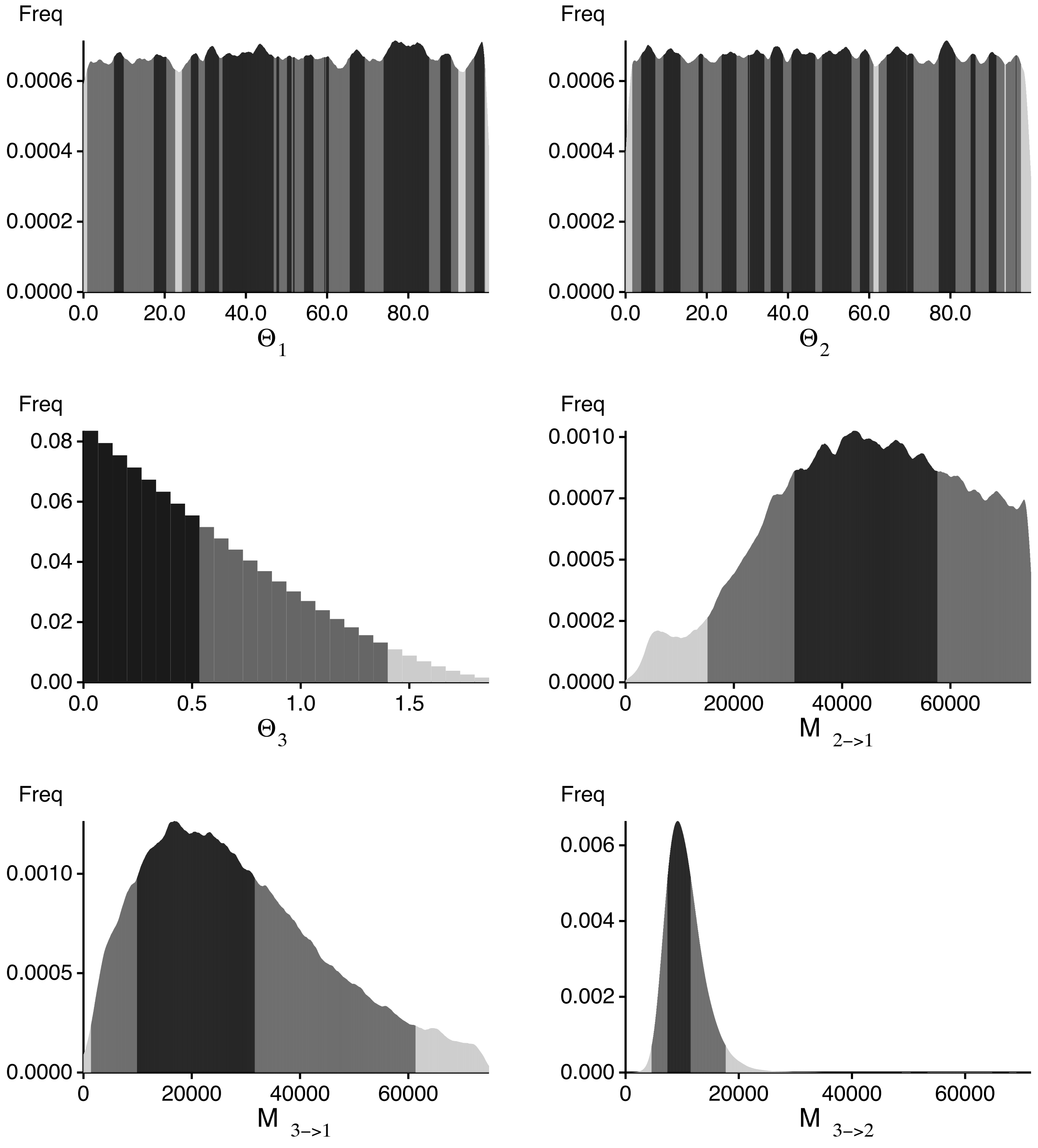

Log-Probability of the data given the model (marginal likelihood)

Use this value for Bayes factor calculations:
BF = Exp[ln(Prob(D I thisModel) - ln(Prob(D I otherModel)
or as LBF = 2 (ln(Prob(D I thisModel) - ln(Prob(D I otherModel))
shows the support for thisModel]

Method	ln(Prob(DIModel))	Notes
Thermodynamic integration	-3098.731465	(1a)
	-2977.257640	(1b)
Harmonic mean	-2516.958060	(2)

(1a, 1b and 2) is an approximation to the marginal likelihood, make sure the program run long enough!
(1a, 1b) and (2) should give a similar result, (2) is considered more
crude than (1), but (1) needs heating with several well-spaced chains,
(1b) is using a Bezier-curve to get better approximations for runs with low number
of heated chains

Acceptance ratios for all parameters and the genealogies

Parameter	Accepted changes	Ratio
Θ_1	625623/625623	1.00000
Θ_2	624206/624206	1.00000
Θ_3	625802/625802	1.00000
$M_{2->1}$	625069/625069	1.00000
$M_{3->1}$	624934/624934	1.00000
$M_{3->2}$	624676/624676	1.00000
Genealogies	488992/2499908	0.19560

MCMC-Autocorrelation and Effective MCMC Sample Size

Parameter	Autocorrelation	Effective Sampe Size
Θ_1	0.00873	49134.86
Θ_2	0.00044	49956.21
Θ_3	0.37825	22555.60
$M_{2->1}$	0.51426	16038.66
$M_{3->1}$	0.47824	17647.93
$M_{3->2}$	0.47263	17905.86
Ln[Prob(D|G)]	0.52423	15606.92
